# Post-Translational Modifications and Diastolic Calcium Leak Associated to the Novel RyR2-D3638A Mutation Lead to CPVT in Patient-Specific hiPSC-Derived Cardiomyocytes

**DOI:** 10.3390/jcm7110423

**Published:** 2018-11-08

**Authors:** Ivana Acimovic, Marwan M. Refaat, Adrien Moreau, Anton Salykin, Steve Reiken, Yvonne Sleiman, Monia Souidi, Jan Přibyl, Andrey V. Kajava, Sylvain Richard, Jonathan T. Lu, Philippe Chevalier, Petr Skládal, Petr Dvořak, Vladimir Rotrekl, Andrew R. Marks, Melvin M. Scheinman, Alain Lacampagne, Albano C. Meli

**Affiliations:** 1 Department of Biology, Faculty of Medicine, Masaryk University, Brno 62500, Czech Republic; acimovic.ivana@gmail.com (I.A.); a.salykin@gmail.com (A.S.); pdvorak@med.muni.cz (P.D.); vrotrekl@med.muni.cz (V.R.); 2 Department of Internal Medicine, Cardiology Division/Cardiac Electrophysiology Section and Department of Biochemistry and Molecular Genetics, American University of Beirut Faculty of Medicine and Medical Center, Beirut 1107 2020, Lebanon; rifaatmarwan@hotmail.com; 3 NeuroMyoGène Institute, University of Claude Bernard Lyon 1, 69100 Villeurbanne, France; adrien.moreau@outlook.com (A.M.); philippe.chevalier@chu-lyon.fr (P.C.); 4 PhyMedExp, INSERM, University of Montpellier, CNRS, 371 Avenue du Doyen G. Giraud, 34295 Montpellier CEDEX 5, France; yvonne.sleiman@etu.umontpellier.fr (Y.S.); monia.souidi@etu.umontpellier.fr (M.S.); sylvain.richard@inserm.fr (S.R.); 5 Department of Physiology and Cellular Biophysics, Clyde and Helen Wu Center for Molecular Cardiology, Columbia University College of Physicians and Surgeons, New York, NY 10032, USA; sr372@columbia.edu (S.R.); arm42@columbia.edu (A.R.M.); 6 CEITEC, Masaryk University, Brno 62500, Czech Republic; pribyl@nanobio.cz (J.P.); skladal@chemi.muni.cz (P.S.); 7 CRBM, CNRS, University of Montpellier, 34293 Montpellier, France and University ITMO, St Petersburg 197101, Russia; andrey.kajava@crbm.cnrs.fr; 8 Department of Cardiology, College of Physicians and Surgeons of Columbia University, New York, NY 10032, USA; dr.jon.lu@gmail.com; 9 International Clinical Research Center, St. Anne’s University Hospital, Brno 60200, Czech Republic; 10 San Francisco Medical Center, University of California, San Francisco, CA 94115, USA; scheinman@medicine.ucsf.edu

**Keywords:** ryanodine receptor, CPVT, hiPSC-derived cardiomyocytes, calcium, β-adrenergic receptor blockade, flecainide, post-translational modifications

## Abstract

Background: Sarcoplasmic reticulum Ca^2+^ leak and post-translational modifications under stress have been implicated in catecholaminergic polymorphic ventricular tachycardia (CPVT), a highly lethal inherited arrhythmogenic disorder. Human induced pluripotent stem cells (hiPSCs) offer a unique opportunity for disease modeling. Objective: The aims were to obtain functional hiPSC-derived cardiomyocytes from a CPVT patient harboring a novel ryanodine receptor (RyR2) mutation and model the syndrome, drug responses and investigate the molecular mechanisms associated to the CPVT syndrome. Methods: Patient-specific cardiomyocytes were generated from a young athletic female diagnosed with CPVT. The contractile, intracellular Ca^2+^ handling and electrophysiological properties as well as the RyR2 macromolecular remodeling were studied. Results: Exercise stress electrocardiography revealed polymorphic ventricular tachycardia when treated with metoprolol and marked improvement with flecainide alone. We found abnormal stress-induced contractile and electrophysiological properties associated with sarcoplasmic reticulum Ca^2+^ leak in CPVT hiPSC-derived cardiomyocytes. We found inadequate response to metoprolol and a potent response of flecainide. Stabilizing RyR2 with a Rycal compound prevents those abnormalities specifically in CPVT hiPSC-derived cardiomyocytes. The RyR2-D3638A mutation is located in the conformational change inducing-central core domain and leads to RyR2 macromolecular remodeling including depletion of PP2A and Calstabin2. Conclusion: We identified a novel RyR2-D3638A mutation causing 3D conformational defects and aberrant biophysical properties associated to RyR2 macromolecular complex post-translational remodeling. The molecular remodeling is for the first time revealed using patient-specific hiPSC-derived cardiomyocytes which may explain the CPVT proband’s resistance. Our study promotes hiPSC-derived cardiomyocytes as a suitable model for disease modeling, testing new therapeutic compounds, personalized medicine and deciphering underlying molecular mechanisms.

## 1. Introduction

Catecholaminergic polymorphic ventricular tachycardia (CPVT) is characterized by episodic syncope under stress conditions and is substantially responsible for sudden cardiac death (SCD) during exercise or acute emotions in the absence of structural cardiac abnormalities. CPVT-related mutations have been associated with mutations affecting the cardiac ryanodine receptor (*RYR2*), calsequestrin (*CASQ2*), triadin (*TRDN*) and calmodulin (*CALM1* and *CALM2*) genes [1].

Investigating the basic underlying mechanisms leading to CPVT has faced difficulties and controversies due to limited models. The emergence of human induced pluripotent stem cells (hiPSCs) allows working with patient-specific cells [2,3].

Here, we report a 25-year-old woman with a novel RyR2 mutation and a history of repeated syncopal episodes after exercise. She was diagnosed with a CPVT syndrome with inappropriate response to metoprolol but responsive to flecainide. Performed genetic analysis revealed a novel RyR2-D3638A mutation located in the highly conserved hot-spot region forming the central domain that transduces the conformational changes [4].

We hypothesized that this mutation is linked to CPVT syndrome, which could be modeled in the dish using patient-specific hiPSC-derived cardiomyocytes (CMs). Thus, we reprogrammed the proband’s dermal fibroblasts into hiPSCs and differentiated the CPVT-hiPSC clones into hiPSC-CMs. We found that CPVT hiPSC-CMs have abnormal contraction force and beat rate associated with electrical disturbances and sarcoplasmic reticulum (SR) Ca^2+^ leak. The RyR2-D3638A mutation causes 3D conformational defects and remodeling including PKA hyperphosphorylation and PP2A and Calstabin2 depletions, which could explain the inappropriate response to metoprolol. In agreement with the clinical observations, we showed that treating CPVT hiPSC-CMs with metoprolol does not prevent these abnormalities while flecainide or stabilizing the RyR2 closed state with a Rycal compound do. We propose a new molecular mechanism to explain why some of the CPVT syndromes do not respond well to ß-blockers.

For the first time, using patient-specific hiPSC-CMs, this study made the link between the 3D conformational consequences of a novel CPVT RyR2 single-point mutation and the resulting RyR2 post-translational modifications associated to SR Ca^2+^ leak, aberrant biophysical properties and inappropriate drug responses. This work suggests the possibility to model some relevant features of cardiac diseases and screen new drugs on hiPSC-CMs.

## 2. Methods

### 2.1. Clinical Evaluation

We obtained a detailed medical history with particular emphasis on syncope, cardiac arrest or symptoms during exertion or emotional stress; medical records, including cardiac evaluation, and a blood sample for genomic DNA extraction. Treadmill stress tests were performed. The diagnosis of CPVT was based on the criteria, which, in short, required stress-induced reproducible ventricular arrhythmias in patients with a normal resting ECG and no detectable structural heart abnormalities. The diagnosis of CPVT was made either on a clinical basis or after identification of a mutation in the *RyR2* gene.

### 2.2. Genetic Studies

Genomic DNA was extracted from whole blood using standard methods. The RyR2 coding regions were amplified using polymerase chain reaction (PCR) and analyzed by denaturing high-performance liquid chromatography.

### 2.3. Cardiac Differentiation

All subjects gave their informed consent for inclusion before they participated in the study. The study was conducted in accordance with the Declaration of Helsinki, and the protocol (10-02214) was approved by the Ethics Committee of the UCSF Medical Center (San Francisco, CA, USA). Healthy control (HC-) and CPVT-hiPSCs as well as hESCs were differentiated into CMs using both, the 2D (monolayer) protocol and 3D (embryoid body (EB)-based) protocol as previously published [5,6]. A detailed Methods section can be found in the Online Data Supplement.

## 3. Results

### 3.1. Clinical Characterization and In Silico Modeling

A 25-year-old athletic woman presented for evaluation in 2005 in the cardiac electrophysiology clinic (UCSF Medical Center, San Francisco, CA, USA) after several episodes of exercise-induced syncope. Episodes occur during strenuous exercise, usually running, but have occurred with bicycling and on a mechanical bull. Typical episodes include a brief prodrome of dizziness without palpitations or chest pain, followed by loss of consciousness and spontaneous recovery in less than one minute. The patient’s first episode occurred at the age of 18 in 1998. From 1998 to 2005, she reported one episode per year, each with strenuous exercise. Her medication list includes oral contraceptive pills. Her parents are alive and well, she has a brother who died suddenly and was attributed to asthma. Four other brothers are in good health. She had a paternal second cousin with aborted SCD after exercise. She has no history of substance abuse. Her physical exam is normal.

**Figure 1:**
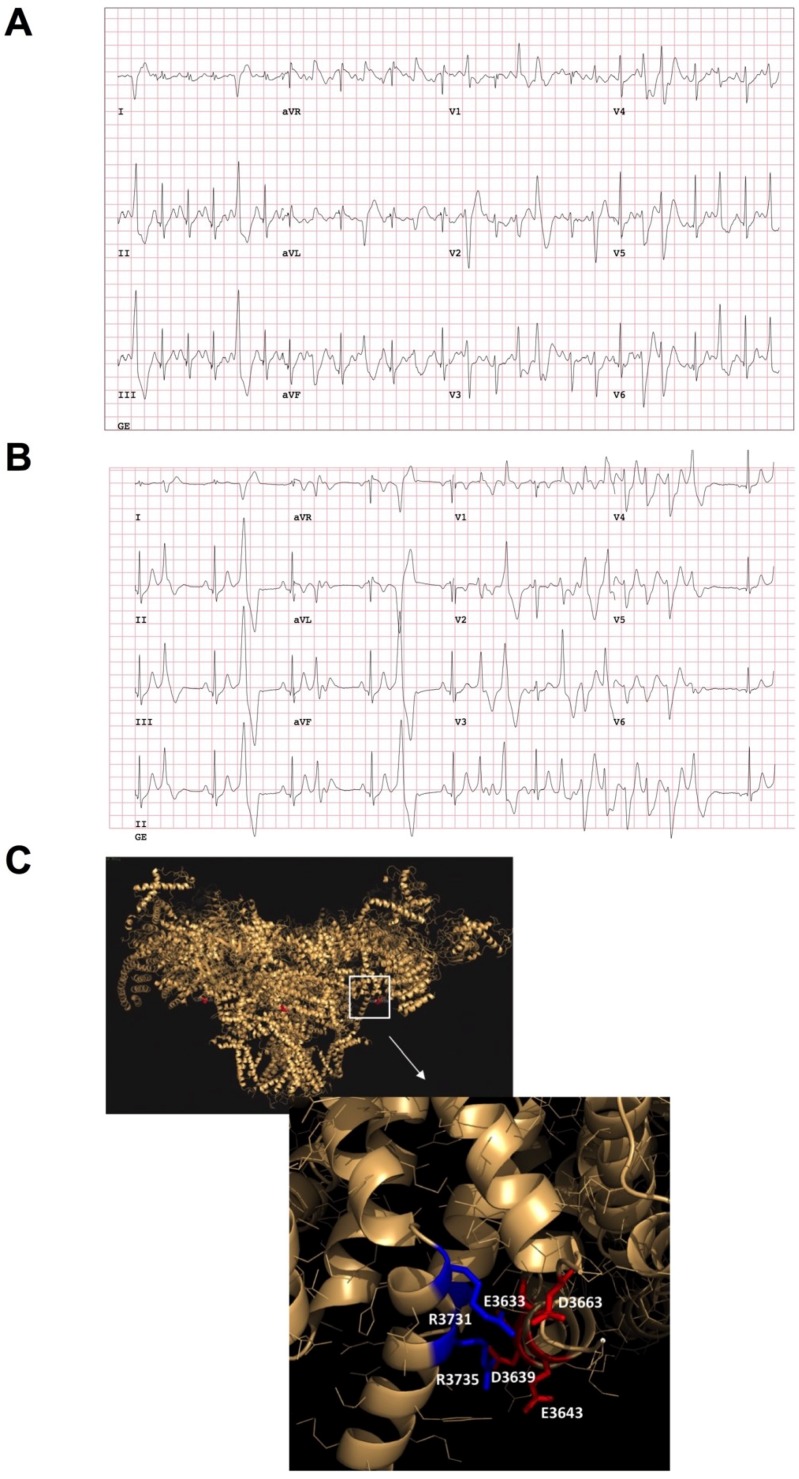
Clinical characterization of the catecholaminergic polymorphic ventricular tachycardia (CPVT) patient and in silico modeling. (**A**) An exercise treadmill test showed a normal QT response to exercise and in recovery, singlet premature ventricular contractions (PVCs) and occasional couplets occurred with exercise with multifocal non-short-coupled PVCs (predominantly RBBB, inferior axis). (**B**) An exercise treadmill test when the proband was treated with metoprolol (METO) (predominantly RBBB, Inferior axis). (**C**) RyR2-D3638A mutation location (red dot, D3639A on the pig amino-acid sequence) on the pig 3D ryanodine receptor (RyR2) resolved structure [4] using MacPyMOL (version 1.7.4.5) and the available PDB file 5goa.cif. This mutation is located in the RyR2 central core domain. In silico modeling analysis revealed that the RyR2-D3638A mutation is located on the surface of a contact between two alpha-helices, via positively charged side chain clusters (K3698, R3731 and R3735) on one side and negatively charged side chains (E3633, E3638, D3639, D3663) on the other side.

Her baseline electrocardiogram (ECG) in 2005 shows sinus bradycardia but is otherwise normal. She had a transthoracic echocardiogram that showed a small patent foramen ovale, minimal shunt with normal systolic and diastolic function. A 24-h ECG Holter showed multifocal premature ventricular contractions (PVCs) with several 3–4 beat runs of non-sustained ventricular tachycardia (NSVT) and an overall PVC burden of <1%. An exercise treadmill test showed a normal QT response to exercise and in recovery, single PVCs and occasional couplets occurred with exercise with multifocal non-short-coupled PVCs (predominantly shape of RBBB, Inferior axis) as shown in Figure 1A. A Tilt table test reproduced the patient’s lightheadedness but not her syncope. A vasodepressor response was observed without bradycardia. An electrophysiology study showed no PVCs and no inducible tachyarrhythmia with aggressive extrastimulation on and off of isoproterenol.

The β-blocker metoprolol (50 mg tablet orally twice daily) was initiated for empiric suppression of PVCs and lifestyle changes were recommended to mitigate vasodepressor response. The patient declined any additional diagnostic testing at that time and described significant baseline fatigue and impairment of exercise tolerance on the β-blocker. A treadmill revealed polymorphic, bidirectional NSVT during recovery from exercise, reflecting an inadequate response and resistance to metoprolol therapy for CPVT (Figure 1B). The heart rates both, at rest and with peak exercise, were reduced proving an effect of the β-blocker, which excluded pharmacokinetic factors. However, due to the onset of ventricular arrhythmias, the test was terminated and the maximal heart rate with β-blocker was not achieved. Metoprolol was eventually discontinued by the patient. She continued to do strenuous physical exercise. In 2009, the patient’s brother with “asthma” was found dead. Postmortem showed no structural disease. Other siblings of Irish descent underwent cardiac testing. By report, one sibling had a baseline ECG with “possible Brugada pattern” but procainamide challenge was negative. No other abnormalities were identified in first-degree family members. The patient had several additional episodes of syncope from 2009 to 2011 when she presented to the cardiac electrophysiology clinic. She was started on flecainide (100 mg tablet orally twice daily) and her arrhythmias were controlled (Figure S1A). She failed the exercise portion of the pre-implantation QRS-T morphology screening of subcutaneous cardioverter-defibrillator and then decided she did not want to have a cardioverter-defibrillator implanted. She subsequently resumed vigorous exercise and completed an Ironman Triathlon with swimming, biking, and running (2015) without any symptoms on flecainide.

Her ECG showed normal sinus rhythm and normal QTc in light of a family history of SCD, exercise-induced symptoms, multifocal PVCs and borderline QTc. Genetic testing was carried out for LQTS and CPVT. *KCNQ1*, *KCNH2*, *SCN5A*, *ANK2*, *KCNE1*, *KCNE2*, *KCNJ2*, *CACNA1C*, *CAV3*, and *SCN4B* were negative for mutations associated with LQTS. All genes checked (including the CPVT2-related gene *CASQ2*) were normal beside a novel heterozygous point mutation of the *RYR2*-c.10913A>C (based on *RYR2* reference sequence NM_001035.2) (Figure S1B). This mutation results in an aspartic acid to alanine substitution at the 3638th position (p.Asp3638Ala or D3638A) of the RyR2 protein. Protein sequence alignment revealed that the aspartic acid is conserved among human, mouse, rat, pig and dog, suggesting its importance in the RyR2 structure/function relationship (Figure S2). The recent 3D structure of RyR2 confirms the importance of D3638A located in the central domain, which likely senses the open and closed conformational changes [4]. The RyR2-D3638A mutation is within a hot-spot region associated with cardiac disorders [7]. We performed in-silico analysis and modeling, based on the recent RyR2 3D structure [4], and found that this single-point mutation is located on the surface that is rich in negatively charged residues and in front of the other surface that is rich in positively charged residues (Figure 1C). The interactions between these charged clusters likely allow the surfaces to be together. At the same time, if small rearrangement of the central domain happens during binding of agonists, it allows these two clusters to still be associated. In fact, by removing Asp3638 (i.e., Asp3639 in pig), the interactions between the charged clusters become weaker which may eventually affect the RyR2 central domain to sense and/or transduce the conformational changes.

### 3.2. Reprogramming of Patient’s Skin Fibroblasts and Characterization of CPVT-hiPSCs

Three CPVT-hiPSC clones (C1.1, C2.2, and C2.5) were successfully generated from patient’s fibroblasts out of 12 clones initially selected, and two of them (C1.1 and C2.2) were used for cardiac differentiation. A hiPSC line (hFiPS1.4) from a healthy subject (age and gender matched) was used as control [8] as well as another control CCTL12 human embryonic stem cell (hESC) line [6]. CPVT-hiPSC clones exhibited undifferentiated phenotype and morphology (Figure S3A), shown by alkaline phosphatase activity (Figure S3B), and expression of pluripotency markers (Figure S3C). Sequencing analysis showed that heterozygous *RYR2*-c.10913A>C (p.D3638A) mutation was conserved (Figure S3D). Karyotype analysis of the CPVT hiPSC line revealed normal chromosomal structure (Figure S3E). Expression of gene markers of all three germ layers in the embryoid bodies (EBs) of independent one-month-old control (hFiPS1.4 and CCTL12) and CPVT lines confirmed their pluripotency (Figure S3F).

### 3.3. CPVT hiPSC Differentiate into Functional Cardiomyocytes

HC and CPVT-hiPSC lines were differentiated in CMs as previously published [5,6]. Immunocytochemical staining of 30-day-old CMs after enzymatic dissociation confirmed that both HC and CPVT hiPSC-CMs demonstrated a cardiac phenotype through striated pattern expression of sarcomeric markers, the cardiac troponin T (cTnT) and cardiac α-actinin. When exposed to isoproterenol, HC and CPVT hiPSC-CMs exhibit the PKA-phosphorylated form of the structural cTnI protein (p-cTnI) (Figure S4A). Contracting EB gene expression analysis by semiquantitative RT-PCR revealed the expression of key cardiac markers such as *RYR2*, myosin light chain 2 (*MYL2*), myosin light chain 7 (*MYL7*), alpha-cardiac actin (*ACTC1*), and connexin 43 (*GJA1*) (Figure S4B). There was no difference in cardiac gene expression between HC and CPVT EBs shown by qRT-PCR (Figure S4C).

### 3.4. CPVT-EBs Present Higher Spontaneous Beats and Weaker Contraction Force Response Under Stress

**Figure 2:**
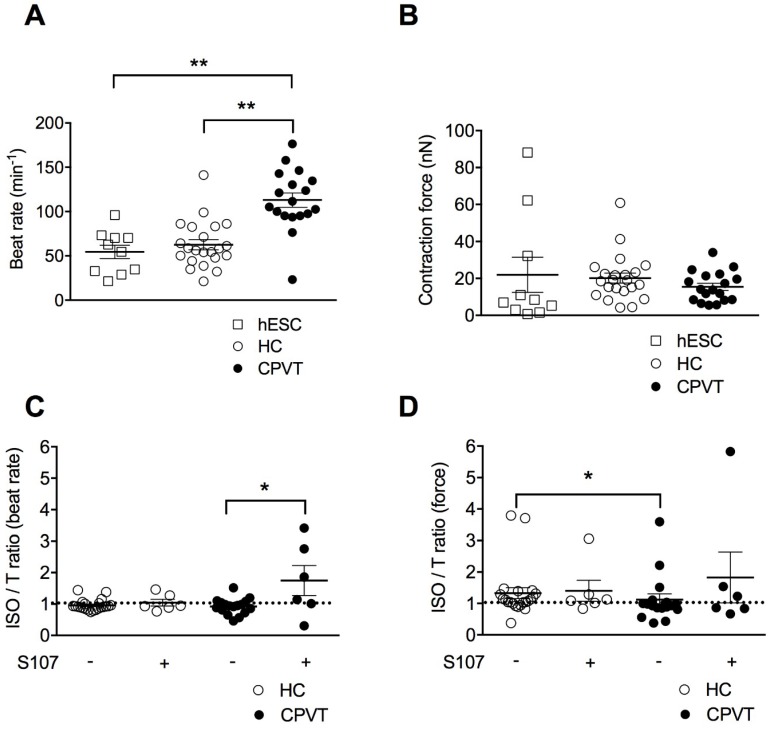
Contractile properties of CPVT human induced pluripotent stem cell-derived embryoid bodies (hiPSC-EBs). (**A**) Scatter plots showing beat rates for control human embryonic stem cell hESC-EBs (hESC), healthy control-EBs (HC) and CPVT-EBs (C2.2, CPVT) at rest. (**B**) Contraction force for hESC-, HC- and CPVT-EBs at rest. (**C**) Ratio of beat rates under 1 µM ISO to beat rates at rest for HC and CPVT ± (indicated by the + or − symbols) 5 µM S107 (overnight). (**D**) Ratio of contraction forces under 1 μM isoproterenol (ISO) to contraction forces at rest for HC and CPVT ± (indicated by the + or − symbols) 5 μM S107 (overnight). The dotted line indicates the ratio threshold. The number of experiments varies from 6 to 22 for each scatter plot. Data are presented with mean ± SEM. * *p* < 0.05; ** *p* < 0.01.

Sinus bradycardia and abnormal cardiac contraction have previously been reported in CPVT patients [9]. Others and we recently developed a method based on atomic force microscopy (AFM) to quantify the contractile properties of beating EBs at rest and under stress conditions [6,10,11,12]. Thus, we aimed here at characterizing the consequence of the RyR2-D3638A CPVT mutation on the rate and contraction force of homogenous clusters of contracting hiPSC-CMs working as a human 3D cardiac syncytium in vitro [13]. In resting physiological conditions at 37 °C and in absence of sympathetic nervous system, CPVT-EBs harboring RyR2-D3638A mutation exhibited a higher beat rate than the HC-EBs (180% increase, Figure 2A). To reinforce the comparison, we used a second group of control EBs differentiated from a hESC line [6,10]. We observed a similar beat rate for the two control EB groups (hESC- and HC-EBs). Both of them had a lower beat rate than the CPVT-EBs (Figure 2A). When looking at the contraction force, no significant difference was observed between hESC-, HC- and CPVT-EBs (Figure 2B).

CPVT syndrome is related to physical or emotional stress involving the activation of the adrenergic signaling pathway. To mimic the stress conditions, we used isoproterenol (ISO), a non-selective β-adrenergic receptor agonist known for its positive inotropic and chronotropic effect. We measured the contraction force and beat rate as a ratio following ISO application over basal condition and observed a slight negative chronotropic effect in the HC- and CPVT-EBs with no significant difference between the 2 groups (Figure 2C). However, while ISO had a positive inotropic effect in HC-EBs (ratio of 1.33 ± 0.17, *n* = 22), it caused a weaker inotropic effect in CPVT-EBs (ratio of 1.12 ± 0.18, *n* = 17, *p* < 0.05) (Figure 2D), suggesting that CPVT-EBs have a weaker and/or abnormal response to ISO in force contraction. Importantly, we obtained similar results using the hESC-EBs as a second control (Figure S5A,B).

### 3.5. Stabilizing the Closed State of RyR2 Using S107 Improves the Contractile Properties in CPVT-EBs

We next aimed at testing a rycal compound (S107) [14] on the EBs in similar experimental conditions. The rycals are known to stabilize the closed the state of RyR2 and prevent SR Ca^2+^ leak in animal models of CPVT, ischemia/reperfusion and heart failure [15,16,17]. S107 induced a strong significant positive chronotropic effect on CPVT-EBs when compared to untreated CPVT-EBs while no effect on HC-EBs (Figure 2C). We observed no difference in the contraction force (ISO/T ratio of 1.12 ± 0.18, *n* = 17 for CPVT-EBs vs. 1.83 + 0.81, *n* = 6 for S107-treated CPVT-EBs) (Figure 2D). S107-treated HC-EBs had similar beat rate compared to untreated HC-EBs (Figure 2C). S107 treatment did not modify the positive inotropic effect of ISO on HC-EBs (Figure 2D).

### 3.6. The CPVT Mutation D3638A Leads to Abnormal Intracellular Ca^2+^ Release under Stress

CPVT-related RyR2 mutations are known to alter the cardiac intracellular Ca^2+^ handling and the excitation-contraction coupling (ECC) in ventricular and atrial CMs [18]. To further investigate the underlying mechanisms linked with the novel CPVT RyR2-D3638A mutation, we measured the intracellular Ca^2+^ handling properties at the excitation-contraction coupling level in Fluo-4 loaded hiPSC-CMs using confocal laser scanning microscopy.

We first characterized the Ca^2+^ handling properties at rest in HC and CPVT hiPSC-CMs. Several parameters were studied including the maximal amount of Ca^2+^ release during a whole Ca^2+^-transient (i.e., maximal Ca^2+^-transient amplitude), the maximal slope of increasing fluorescence intensity with time (dF/dt_max_), which indicates the rate of RyR2 Ca^2+^ release and the Ca^2+^ amount mobilized upon the transients (i.e., area under the curve or peak area). Aberrant events as systolic double-humped Ca^2+^-transients (called “aberrant Ca^2+^-transients”) and additional diastolic events of low amplitude between the transients (called “diastolic SR leaky events”) were also evaluated.

In basal conditions, HC hiPSC-CMs and CPVT hiPSC-CMs showed similar spontaneous release of Ca^2+^ with no difference in the Ca^2+^-transient maximal amplitude (Figure S6A,B). We observed no particular difference in the occurrence of systolic Ca^2+^-transients and diastolic SR leaky events (Figure S6C,D) and no difference in the rate of RyR2 Ca^2+^ release or in the peak area between HC and CPVT hiPSC-CMs (Figure S6E,F). These results indicate no difference in the major properties reflecting the Ca^2+^ release from the RyR2 channels and might rule out the role of RyR2 in the higher beat rate observed in CPVT-EBs at rest (Figure 2A).

**Figure 3:**
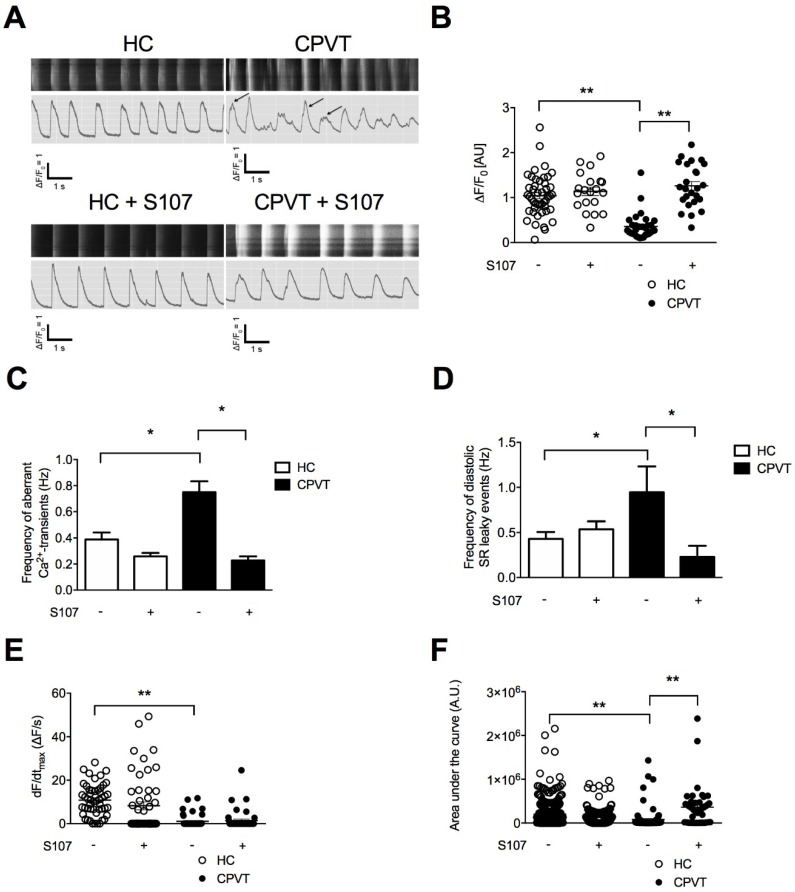
Intracellular Ca^2+^ cycling and SR Ca^2+^ stores in CPVT hiPSC-CMs under stress. (**A**) Display of original line-scan images of Ca^2+^ transients and corresponding tracings of HC and CPVT C2.2 hiPSC-CMs treated with 1 μM ISO together with pacing at 1 Hz (20 V, 0.5 ms duration and 1 ms delay) ± 5 μM S107. Additional and aberrant Ca^2+^ release events are shown with the arrows. (**B**) Scatter plots showing maximal Ca^2+^-transient amplitude in HC (white dots) and CPVT (black dots) hiPSC-CMs under stress conditions ± (indicated by the + or − symbols) treated with 5 μM S107. (**C**,**D**) Bar graphs showing frequency of occurrence of aberrant Ca^2+^-transients (**C**) and diastolic SR leaky events (**D**) in HC and CPVT hiPSC-CMs under stress conditions ± (indicated by the + or − symbols) treated with 5 μM S107. (**E**,**F**) Rate of RyR2 Ca^2+^ release (dF/dt_max_) and area under the curve (peak area) in HC and CPVT hiPSC-CMs under stress conditions ± (indicated by the + or − symbols) treated with 5 μM S107. The number of experiments varies from 20 to 46 for each scatter plot. Data are presented with mean ± SEM. * *p* < 0.05; ** *p* < 0.01.

We next explored the effect of stress by combining ISO and pacing. We found that CPVT hiPSC-CMs exhibit significant lower maximal Ca^2+^-transient amplitude (Figure 3A,B). Significant increases in the frequency of aberrant Ca^2+^-transients during systole and diastolic SR leaky events were observed in CPVT hiPSC-CMs when compared to HC hiPSC-CMs (Figure 3C,D). These results suggested that stress triggers more irregular Ca^2+^-release events in CPVT hiPSC-CMs. Especially, the diastolic SR leaky events are reminiscent of the sub-conductance states of RyR2 mutated channels we previously observed [19]. When looking at the kinetic properties of RyR2 Ca^2+^ release, CPVT hiPSC-CMs exhibit a lower rate of Ca^2+^ release through RyR2 (Figure 3E) and mobilized less Ca^2+^ than the HC hiPSC-CMs (i.e., peak area, Figure 3F) in agreement with the decreased maximal Ca^2+^-transient amplitude (Figure 3A,B). These irregularities under stress in CPVT hiPSC-CMs may consequently impair the ECC and the contraction force as observed in CPVT-EBs (Figure 2D). No significant difference in the fractional Ca^2+^ release or in the time to peak under caffeine (10 mM) between CPVT and HC hiPSC-CMs (Figure S7A–C).

### 3.7. S107 Prevents the SR Ca^2+^ Leak in CPVT hiPSC-CMs and Does Not Affect HC hiPSC-CMs

We next tested whether S107 could also prevent the systolic and diastolic aberrant Ca^2+^ events under stress. CPVT hiPSC-CMs treated with S107 exhibited less systolic and diastolic aberrant Ca^2+^ events than untreated cells, suggesting a stabilizing effect of S107 on RyR2-D3638A channels, a more stable intracellular Ca^2+^ release and a better diastolic phase when the RyR2 channels should be tightly closed (Figure 3A,C,D). S107 was able to increase the maximal Ca^2+^-transient amplitude (Figure 3A,B) and the peak area in CPVT hiPSC-CMs (Figure 3F). It did not change the rate of Ca^2+^ release through RyR2 (Figure 3E). None of those properties were modified in S107-treated HC hiPSC-CMs (Figure 3A–F).

### 3.8. Unlike Flecainide, Metoprolol Does Not Prevent the Abnormal Ca^2+^ Release in CPVT hiPSC-CMs

The clinical observations revealed that the proband had no improvement under metoprolol (METO, selective β_1_-adrenergic receptor blocker) beside a reduced heart rate. However, she had no symptom under flecainide treatment alone. We thus aimed at testing whether the inadequate response to METO and potent effect of flecainide could be recapitulated using CPVT hiPSC-CMs by focusing on the intracellular Ca^2+^ handling properties under stress conditions. We found that none of the studied properties were modified under METO (Figure S8A–F). CPVT METO-treated hiPSC-CMs exhibited aberrant Ca^2+^-transients and diastolic SR Ca^2+^ leak with no difference when compared to non-treated cells (Figure S8A–D). We checked whether CPVT-EBs express similar level of β_1_- and β_2_-adrenergic receptors and found no significant difference compared to HC-EBs (Figure S7D).

**Figure 4:**
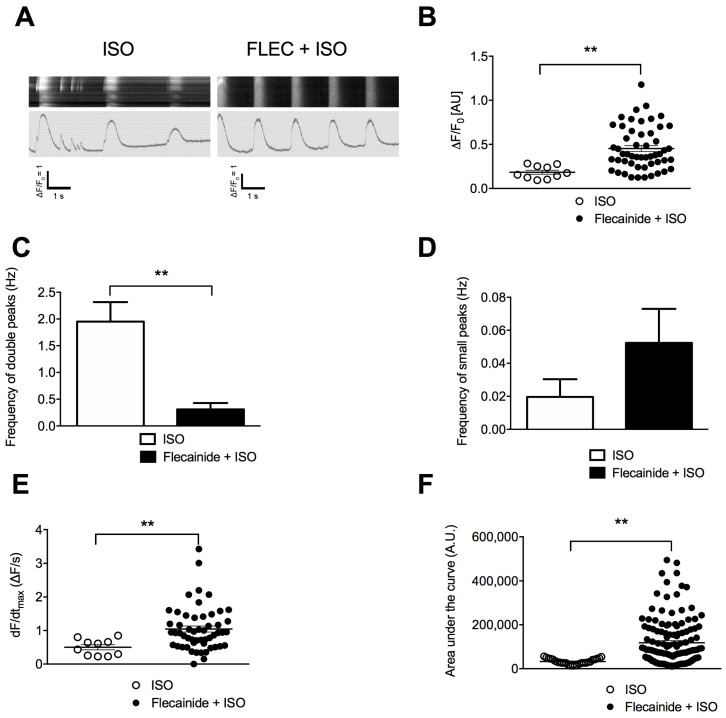
Flecainide (FLEC) partially prevents the aberrant SR Ca^2+^ handling in CPVT hiPSC-CMs. (**A**) Display of original line-scan images of Ca^2+^ transients and corresponding tracings of CPVT (C1.1) hiPSC-CMs after application of ±5 μM flecainide followed by 1 μM ISO. (**B**) Scatter plots showing maximal Ca^2+^-transient amplitude in CPVT hiPSC-CMs under ISO (white dots) and flecainide + ISO (black dots). (**C**) Bar graphs showing frequency of occurrence of aberrant Ca^2+^-transients in CPVT hiPSC-CMs under ISO and flecainide + ISO. (**D**) Frequency of occurrence of diastolic SR leaky events in CPVT hiPSC-CMs under ISO and flecainide + ISO. (**E**) Rate of RyR2 Ca^2+^ release (dF/dt_max_) in CPVT hiPSC-CMs under ISO and flecainide + ISO. (**F**) Area under the curve or peak area in CPVT hiPSC-CMs under ISO and flecainide + ISO. The number of experiments varies from 10 to 55 for each scatter plot. Data are presented with mean ± SEM. ** *p* < 0.01.

Unlike METO, flecainide prior ISO significantly increased the maximal amplitude of the Ca^2+^-transients, rate of Ca^2+^ release as well as peak area (Figure 4). It significantly reduced the frequency of aberrant Ca^2+^-transients, which overall led to more regular Ca^2+^-transients although no change in the frequency of the diastolic SR leaky events (Figure 4A,C,D). 

### 3.9. Electrical Activity Is Affected in CPVT RyR2-D3638A Patient Cells

To get better insight in the electrical activity of HC and CPVT hiPSC-CMs, the patch-clamp technique (current-clamp) was used to study action potentials (AP) parameters. Both spontaneous and elicited AP were studied in the whole-cell configuration. To eliminate nodal-like CMs, cells with an AP duration at 90% of repolarization (APD_90_) shorter than 100 ms were discarded and not analyzed (16% and 27% of cells for HC and CPVT respectively) (Figure S9). As expected, recorded AP mainly demonstrated a typical ventricular-like shape. Indeed, when elicited at 1 Hz, APD_90_ were longer than 280 ms and the mean APD_90_/APD_50_ ratio was 1.8 and 1.6 (with a 95% CI of the mean of 1.5 and 2.1/1.5 and 1.9 for HC and CPVT respectively), indicating the typical plateau phase of ventricular like AP (Figure S9).

**Figure 5:**
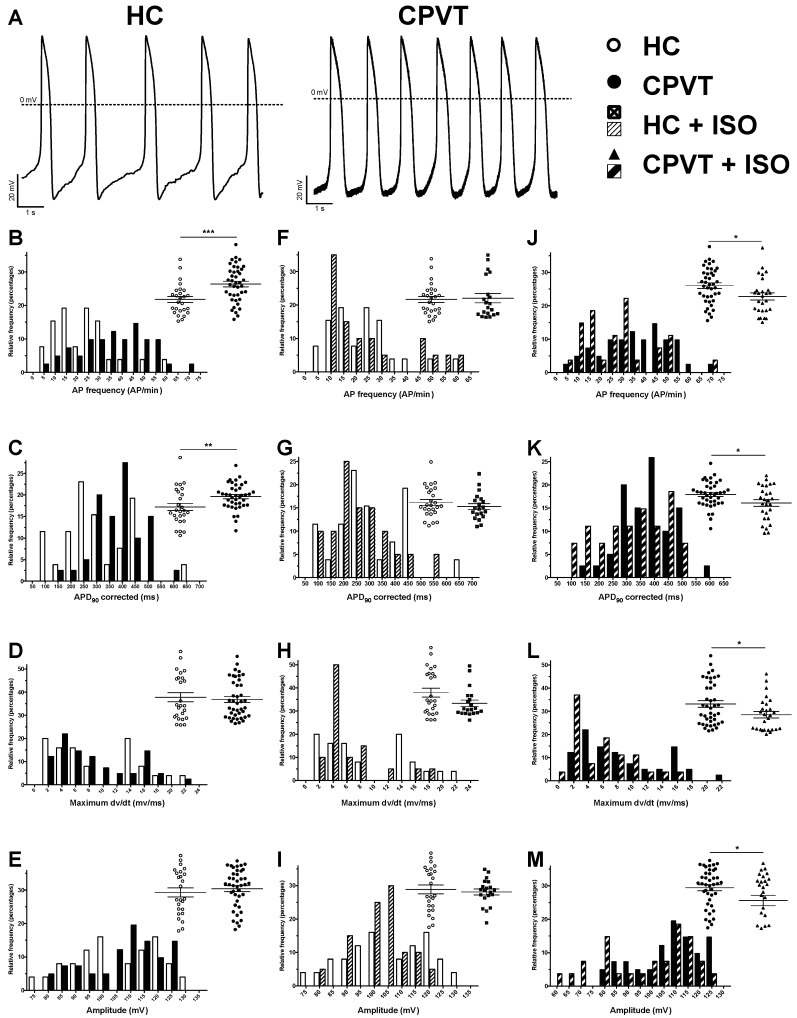
Action potential parameters from spontaneous electrical activity. (**A**) Example of spontaneous action potentials (AP) from HC (left) and CPVT (right) hiPSC-CM recorded using the patch-clamp technique on isolated cardiomyocytes. (**B**–**M**) AP characteristics are studied and were compared between: HC (*n* = 26) and CPVT (*n* = 41) (**B**–**E**), HC (*n* = 26) and HC + ISO (*n* = 20) (**F**–**I**) and CPVT (*n* = 41) and CPVT + ISO (*n* = 29) (**J**–**M**). For each comparison, the frequency distribution is shown with a scatter plot (with mean ± SEM) in inset to provide an accurate view of population distribution. The parameters studied were the spontaneous AP frequencies (**B**,**F**,**J**), the bazett’s corrected APD_90_ (**C**,**G**,**K**), the maximum depolarization speed (maximum dV/dt) (**D**,**H**,**L**) and the AP amplitude (**E**,**I**,**M**). * *p* < 0.05; ** *p* < 0.01, *** *p* < 0.001.

At rest, spontaneous AP from CPVT hiPSC-CMs demonstrated several differences when compared with HC hiPSC-CMs (Figure 5, Tables S1 and S2). Indeed, the spontaneous AP frequency is significantly increased in CPVT cells, in accordance with observations made in AFM experiments (Figure 5, Table S1). As the frequency differs, the APD_90_ was corrected accordingly using the bazett’s formula (APD_90_/square root (1/APfrequency)). As demonstrated by the large right shift of black bars in Figure 5C, the corrected APD_90_ was increased in CPVT hiPSC-CMs (418 ± 29 ms, *n* = 26) when compared to HC hiPSC-CMs (287 ± 20 ms, *n* = 26, *p* < 0.01). In contrast, the depolarization speed, illustrated through the measure of the maximum dV/dt, and AP amplitude were similar in CPVT and HC hiPSC-CMs (Figure 5, Table S1). Of note, the dV/dt_max_ in spontaneous AP appears low in both HC and CPVT (Table S1). This can be explained by the depolarized maximum diastolic potential (MDP) of −66.4 ± 1.4 and −65.6 ± 1.1 mV for HC and CPVT respectively most probably resulting in a voltage gated sodium channels inactivation. hiPSC-CM were then paced at a fixed frequency. To robustly compare AP parameters from HC and CPVT, the MDP was artificially lowered at −80 mV and AP were elicited at 1 Hz. Except for the AP amplitude, AP parameters were confirmed through the 1 Hz pacing of HC and CPVT hiPSC-CMs (Figures S10 and S11, Table S2). In these conditions, the dV/dt_max_ increased when compared to spontaneous activity. Such increased most probably rely on a recovered fraction of voltage gated sodium channels due to the constrained MDP.

To further mimic the consequences of exercise described as an arrhythmic trigger by the patient, HC and CPVT hiPSC-CMs were stimulated at 2 Hz (Figure S12, Table S2). APD_20,50 and 90_ were significantly longer while the maximum dV/dt, the amplitude and the overshoot did not show any differences when compared to HC hiPSC-CMs (Figure S12, Table S2). The effect of changes in stimulation frequency were further evaluated and expressed as percentages of variation. This comparison revealed larger variations between HC and CPVT cells when cells were stimulated at 2 Hz (when compared to variations when cells were stimulated at 1Hz) (Figure S13, Table S3). A likely explanation can be found when comparing variations between 1 and 2 Hz for each hiPSC-CMs line (Figure S13, Table S3). Indeed, variations for CPVT are less pronounced than variations for HC hiPSC-CMs. Taken together, this would indicate more severe electrical phenotype with increased AP frequency.

1 μM of isoproterenol was used to assess the effect of ß-adrenergic stimulation on the electrical membrane activity (Figure 5). None of the parameters were significantly modified in HC hiPSC-CMs, while all values were decreased in CPVT-ISO condition (AP frequency, APD90 corrected, maximum dV/dt and AP amplitude) (Figure 5).

### 3.10. Abnormal Electrical Activity Elicited with ISO Is Rescued with Flecainide and S107

**Figure 6:**
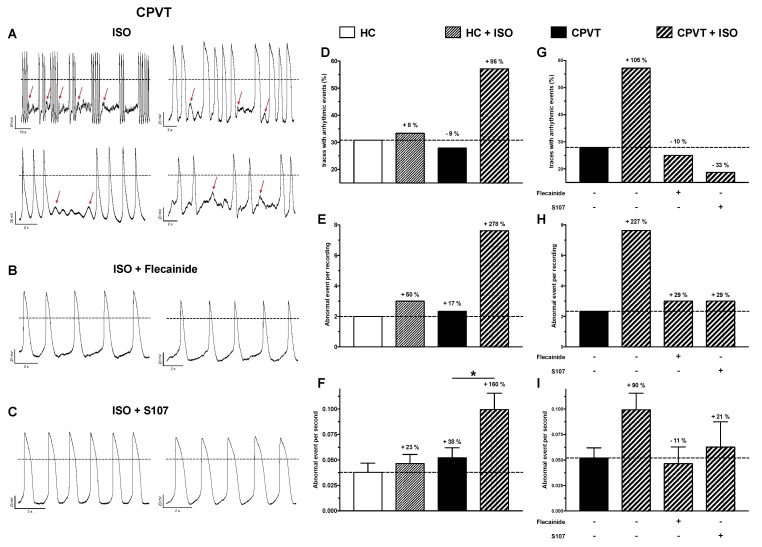
Abnormal electrical activity is elicited by ISO and normalized with flecainide and S107 treatments. (**A**–**C**) Examples of current clamp raw traces showing APs recorded from CPVT hiPSC-CM after treatment with ISO (1 μM) (**A**), flecainide (5 μM) + ISO (**B**) and S017 + ISO (5 μM) (**C**). (**D**–**F**) HC and CPVT abnormal electrical activity is studied with and without ISO (HC: *n* = 26, HC + ISO: *n* = 20, CPVT: *n* = 41, CPVT + ISO: *n* = 29). Percentages of variations are indicated in comparison with HC. (**G**–**I**) The effect of flecainide (*n* = 12) and S107 (*n* = 16) pre-treatment (indicated by the + in contrast to -) on CPVT hiPSC-CM is also studied. Abnormal electrical activity is evaluated through the measure of the number of trace with aberrant events (**D**,**G**), the measure of abnormal event in each recording showing at least 1 abnormal event (**E**,**H**) and their frequency in Hz (**F**,**I**). * *p* < 0.05.

Besides the AP parameters, the abnormal electrical events were also studied (Figure 6). Such events are determined as early or delayed after-depolarizations as depicted Figure 6A. Without treatments, the number of CPVT cells with aberrant events is slightly decreased when compared with HC hiPSC-CMs, however, their frequency is increased by 38% (events per second of recording) (Figure 6D–F). While the addition of ISO has low effect on HC electrical aberrant events, all measured parameters are drastically increased in CPVT hiPSC-CMs (Figure 6D–F). Indeed, when compared to non-treated HC hiPSC-CMs, CPVT cells demonstrated 86% increase in traces with aberrant electrical activity, a 278% and a 160% respective increase in the number of abnormal events per recording and in their frequency (Figure 6D–F).

Patient treatment was mimicked with addition of 5 μM flecainide before ISO challenging (Figure 6G–I). This addition normalized the number of traces with abnormal events, their frequency and drastically reduced the number of abnormal events per recording (Figure 6G–I). The addition of flecainide even normalized the spontaneous AP frequency (in Hz): 0.39 ± 0.04 (*n* = 26, HC), 0.61 ± 0.04 (*n* = 41, CPVT), 0.44 ± 0.05 (*n* = 29, CPVT-ISO) and 0.36 ± 0.06 (*n* = 12, CPVT flecainide-ISO).

To confirm the benefit of reducing the diastolic Ca^2+^ leak, CPVT hiPSC-CMs were also subjected to S107 treatment before ISO challenging (Figure 6G–I). The addition of S107 clearly improved abnormal electrical activity reducing the number of traces with aberrant events, their number per recording and their frequency (Figure 6G–I) when compared with ISO condition.

### 3.11. Post-Translational Modifications Are Associated with the CPVT RyR2-D3638A Channels

SR Ca^2+^ leak through RyR2 has been associated with post-translational modifications of the RyR2 macromolecular complex including PKA-phosphorylation and Calstabin2 depletion [20]. We explored the RyR2 macromolecular complex by co-immunoprecipitation in HC and CPVT cell lysates under resting and stress conditions. We evaluated the amount of Calstabin2 bound to RyR2 and the level of RyR2 PKA phosphorylation at its specific site S2809 and incubated with flecainide (FLEC), metoprolol (METO) and S107. We also explored some of the key proteins responsible for reversing the phosphorylation modifications such as type 2 protein phosphatase (PP2A) and spinophilin (PP1 anchoring protein). 

**Figure 7:**
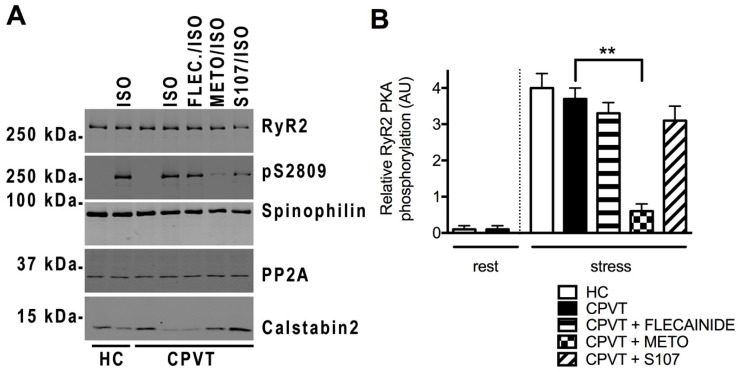

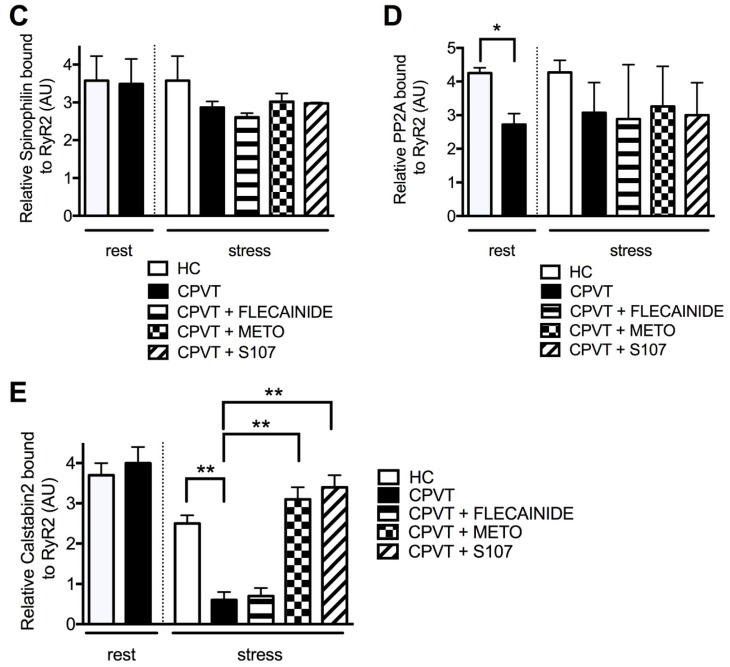
PKA-phosphorylated CPVT RyR2-D3638A mutant channels are depleted in Calstabin2 which is prevented by S107 but not by flecainide. (**A**) Representative immunoblot bands of HC and CPVT lysates (c2.2) at rest and under stress (1 μM ISO application) for total RyR2, PKA-phosphorylated RyR2 at S2809 site, spinophilin, PP2A and Calstabin2. In stress conditions, CPVT cells were also either pretreated with 5 μM flecainide (FLEC.), or 5 μM metoprolol (METO) or 5 μM S107 (**B**–**E**) Bar graphs showing data normalization from immunoblots for the relative RyR2 PKA phosphorylation, spinophilin, PP2A and Calstabin2 bound to RyR2 in HC and CPVT at rest and under stress and in presence of flecainide, METO and S107. For each condition, *n* = 3 to 8 independent experiments. * *p* < 0.05; ** *p* < 0.01.

At rest, we found no PKA phosphorylation and similar Calstabin2 level associated to RyR2 in HC and CPVT hiPSC-CMs. Under stress conditions, CPVT hiPSC-CMs were significantly more depleted in Calstabin2 (416% less) from the RyR2 macromolecular complex than HC hiPSC-CMs with similar level of RyR2 PKA-phosphorylated at its Ser2809 (Figure 7A,B,E). Surprisingly, we found significantly less PP2A in CPVT (2.72 ± 0.32, *n* = 6) compared to HC (4.25 ± 0.16, *n* = 5; *p* < 0.05) and similar spinophilin bound to the RyR2 macromolecular complex in resting conditions (Figure 7A,C,D).

We next tested whether the effects of flecainide, METO and S107 under stress were associated with RyR2 post-translational modifications in CPVT cells. Interestingly, flecainide treatment did not prevent the PKA phosphorylation at Ser2809 and Calstabin2 depletion while METO reduced both (517% decrease for pS2809), likely by selectively blocking the ß1-adrenergic receptors. S107 treatment prevented Calstabin2 depletion from the RyR2 macromolecular complex in CPVT hiPSC-CMs (Figure 7A,C,E). There was no particular difference in the amount of spinophilin and PP2A bound to CPVT RyR2 under stress (Figure 7A,C,D).

## 4. Discussion

Despite several studies modeling CPVT and testing drugs in the dish [13,21,22,23,24], there is no clear understanding of the molecular mechanisms underlying this syndrome in human. In fact, several mechanisms have been suggested using animal models and recombinant strategies and none of them really proven in human. Previously, we demonstrated that RyR2 single-point mutations linked with CPVT exhibit cytosolic Ca^2+^ hypersensitivity and diastolic leak associated with Calstabin2 depletion under stress [19]. The present study markedly extends these earlier studies by showing that in patient-specific CMs, the CPVT RyR2-D3638A mutation causes a macromolecular complex remodeling including depletion of Calstabin2 under stress, indicating that such RyR2 post-translational modifications are a mechanism in human CMs, which we believe to be novel to explain stress-induced ventricular arrhythmias and pharmacological responses observed clinically including inappropriate response to standard β-adrenergic receptor blockade.

We generated patient-specific hiPSC-CMs from a proband harboring a novel single point mutation RyR2-D3638A located in a highly conserved central domain and associated with CPVT syndrome under stress conditions. Consistently with the clinical observations, we found that the contractile, electrical and SR Ca^2+^ handling properties were abnormal under stress. These aberrations were not prevented by the standard β-adrenergic receptor blockade (Metoprolol) while the flecainide and S107 prevented them. Most important, and among the several CPVT disease modeling studied, we found a novel RyR2 macromolecular complex remodeling for the first time using hiPSC-CMs which could explain the pharmacological responses observed clinically.

### 4.1. Insights from the Structure-Function Aspects

The RyR2-D3638A mutation is within a hot-spot region associated with cardiac disorders [7]. The highly-conserved Asp residue mutated in this study may be of high importance in the RyR2 structure/function relationship. Based on the recently resolved RyR1 structure [25] a salt bridge between D3671 and R3769 may exist in RyR1. The negatively charged RyR2-D3638 may for a salt bridge with the positive R3730 (RyR2 equivalent of RyR1 R3769). Similar functional defects would be expected due to the R3730 mutation. The recent 3D structure of RyR2 by cryo-EM highlights the central domain in which the D3638 is included. The central domain is constituted of U-motifs involved in rearrangements driving the closed and open states of RyR2 [4]. The in-silico analysis and modeling revealed that this single-point mutation is located on the rich surface of negatively charged residues facing a surface rich in positively charged residues. These potential interactions might be altered by the D3638A mutation causing weaker charge clusters which may eventually affect the RyR2 central domain to sense and/or transduce the conformational changes.

Therefore, the D3638A mutation could impair the conformational changes upon agonists such as Ca^2+^. In this context, the diastolic SR Ca^2+^ leak observed in CPVT hiPSC-CMs could be explained by an unstable closed state of the RyR2-D3638A mutant that is normally tightly regulated by low diastolic Ca^2+^. The RyR2-D3638A mutation does not appear to affect the RyR2 leucine/isoleucine zipper motifs (LZ motifs) for the anchoring proteins AKAP, spinophilin and PR130 which allow the interactions with PKA, PP1 and PP2A, respectively [26]. Indeed, they are rather located in the cytoplasmic peripheral structures SPRY, P1 and P2 domains.

### 4.2. Modeling of CPVT Syndrome Using hiPSC-CMs in the Dish

Our previous studies showed no difference in the cardiac faith for several human pluripotent stem cell lines upon the differentiation protocol we developed [6,10]. Based on the patch-clamp experiments, we worked with a population mostly composed of ventricular-like hiPSC-CMs. Using an AFM-based method we developed [6,10], we here revealed aberrant beat rate and contraction force in CPVT 3D beating clusters during resting and stress conditions, respectively. At rest, CPVT-EBs exhibited increased beat rate in correlation with increased frequency for spontaneous depolarization observed in CPVT hiPSC-CMs although the intracellular SR Ca^2+^ handling properties were similar to those in HC hiPSC-CMs. One experimental limit is that the AFM-based experiments were performed at 37 °C while the patch-clamp and Ca^2+^ imaging were done at room temperature (~22 °C). Therefore, the interpretation of these results should be taken with caution. It should be noted that CPVT cells exhibited depleted PP2A from the RyR2 channels which overall may cause an aberrant ECC independent of an obvious leaky RyR2 at rest in CPVT cells.

In agreement with the clinical observations, our results indicated that the RyR2-D3638A mutation in CPVT hiPSC-CMs leads to an abnormal Ca^2+^ release under stress conditions by decreasing the Ca^2+^ transient amplitude and increasing the occurrence of both, the systolic and diastolic aberrant Ca^2+^ events. These features suggested that the RyR2-D3638A mutation triggers systolic aberrant Ca^2+^-transients and a diastolic SR Ca^2+^ leak which eventually impairs the contraction force in the CPVT-EBs. Interestingly, the diastolic SR Ca^2+^ leak we observed in CPVT hiPSC-CMs are reminiscent of the leaky sub-conductance states of CPVT RyR2 channels previously observed by our group [19], although we did not measure their amplitude. Our results ruled out a lower SR Ca^2+^ content in CPVT hiPSC-CMs under stress conditions as previously suggested in CPVT [21,27].

Although the CPVT syndrome is clinically revealed by ventricular arrhythmias, studies revealed that nodal [28] and atrial CMs [15] are also affected by CPVT mutations leading to intracellular Ca^2+^ handling abnormalities and contributing to sinoatrial node dysfunction and atrial fibrillation. The patch-clamp experiments revealed clear alteration in both spontaneous and paced electrical activity. At rest, the non-altered dV/dt suggested a normal cellular depolarization step and normal tissue conduction while the AP lengthening constitutes a favorable substrate for arrhythmias. Increasing the stimulation frequency at 2 Hz worsen AP parameters defect, consistently with the observed increased in arrhythmic events in the patient during exercise. Similarly, adding ISO to mimic stress condition notably decreased both depolarization speed and AP amplitude further amplifying arrhythmogenic potential. Interestingly, strong consistency can be observed comparing electrical and contractile CPVT functions. Our present study brings new evidences that heterogeneous populations of patient-specific CPVT hiPSC-CMs commonly exhibit stress-induced abnormal intracellular Ca^2+^ handling and contractile properties.

### 4.3. The Inadequate Β-Blocker Response and Potent Effects of S107 and Flecainide

The clinical results revealed that VTs during exercise are not prevented by metoprolol treatment in the proband harboring the RyR2-D3638A mutation. However, the heart rate both at rest and with peak exercise were reduced. This observation excluded an ultra-rapid metabolizing status notably by the liver enzyme CYP2D6 [29] and proved the metoprolol effect. We evaluated the effect of metoprolol on the Ca^2+^-release through RyR2 in the CPVT hiPSC-CMs and observed that the leaky phenotype through RyR2 was not prevented properly. We showed that metoprolol partially reduced the RyR2 PKA phosphorylation and Calstabin2 depletion. Interestingly, metoprolol treatment caused a (non-significant) trend to reduced SR diastolic leaky events while flecainide did not. Therefore, reducing Calstabin2 depletion through reduced RyR2-PKA phosphorylation leads to reduced leaky events in CPVT hiPSC-CMs. While these evidences are, for the first time, observed in human CMs, they are in agreement with our previous study showing that Calstabin2 depletion through PKA phosphorylation, a consequence of the ß-adrenergic stimulation, is associated to leaky RyR2 channels under stress in agreement with the clinical observation of CPVT syndrome [19]. These results also correlated with our previous work showing that mice with constitutive RyR2 PKA-phosphorylation (S2808D^+/+^ mice) result in diastolic leak and cardiac dysfunction. Moreover, we showed that reversing RyR2 PKA phosphorylation is an important mechanism of the ß-blockers to prevent cardiac dysfunction [20]. However, although metoprolol may not be the optimal β-blocker for CPVT, it is the first time here that its inadequate response is shown using patient-specific hiPSC-CMs correlating with the clinical observations. Our results not only confirmed that hiPSC-CMs can recapitulate patient-specific drug responses as recently shown [24] but they go beyond by suggesting a novel molecular mechanism of ß_1_-blocker resistance in CPVT in human. At a concentration known to be efficient in CMs [22], we showed that flecainide partially restored the regular intracellular Ca^2+^-release properties in CPVT hiPSC-CMs in stress conditions but did not prevent the diastolic SR Ca^2+^ leak. Flecainide also normalized aberrant event recorded using the patch-clamp technique. The spontaneously increased AP frequency is also corrected under flecainide treatment. Here we revealed for the first time in human CMs, that flecainide does not prevent the pathological Calstabin2 depletion in CPVT cells which is known to be associated to leaky RyR2 channels [15,16,17,19,20,30,31,32,33,34,35]. Does flecainide directly act on RyR2 channel? The molecular mechanisms underlying the potency of flecainide is unclear [36,37,38] and the present results suggest another target than RyR2.

Furthermore, we observed a potent effect of S107 on CPVT hiPSC-CMs by stabilizing the contraction force and the beat rate as well as by preventing SR Ca^2+^ leak under stress conditions. A very recent study also showed a potent effect of S107 in CPVT hiPSC-CMs harboring the RyR2-I4587V mutation [23]. Here, we found for the first time in hiPSC-CMs, that the S107 beneficial effect relies on stabilizing the closed state of RyR2 likely via its stabilizing Calstabin2 partner as previously shown in mice [15,20,35]. Further beneficial S107 effect were observed on spontaneous electrical activity, normalizing aberrant events elicited by ISO. These new findings on human cardiac tissue may provide further pharmacological approaches to determine how these drugs specifically work.

### 4.4. Study Limitations

Our present study is about one patient diagnosed with CPVT harboring the novel RyR2-D3638A single-point mutation. We unfortunately did not have access to other relatives of this patient for further phenotypic and genomic analysis. Identifying new families with similar phenotypes and RyR2 mutations would reinforce the functional consequences of this novel RyR2 mutation. Our study did not include an isogenic control to compare with the RyR2-D3638A mutant. Although the recent genome editing using the CRISPR/Cas9 tool allows to correct single-point mutations, we did not attempt to correct such gene. Instead we used 2 control lines which behaved similarly.

## 5. Conclusions

To conclude, we describe a woman with CPVT syndrome, harboring the novel RyR2-D3638A mutation and responsive to flecainide but not to metoprolol. The RyR2-D3638A mutation leads to 3D conformational defects, aberrant functional properties associated to RyR2 post-translational macromolecular complex remodeling for the first time revealed using CPVT patient-specific hiPSC-derived cardiomyocytes which may explain the CPVT proband’s resistance to metoprolol. We report the first association of CPVT RyR2 mutation in hiPSC-CMs causing post- translational modifications and diastolic calcium leak correlating with the clinical observations. We have identified key novel molecular mechanisms underlying the CPVT syndrome in a patient-specific cell context.

## Supplementary Materials

The following are available online at http://www.mdpi.com/2077-0383/7/11/423/s1. Table S1: Spontaneous AP parameters; Table S2: AP parameters at different pacing; Table S3: Paced AP parameters variations; Figure S1: (**A**) An exercise treadmill test showed the disappearance of VTs when the proband was treated with 100 mg flecainide (predominantly RBBB, Inferior axis). (**B**) DNA sequence chromatogram of the proband showing the heterozygous single nucleotide substitution (indicated by M) adenine (A) to cytosine (C) of the *RyR2* gene nucleotide sequence (RYR2-688, 730A>C). This mutation results in an aspartic acid to alanine substitution at the 3638th position (Asp3638Ala or D3638A) of the RyR2 protein; Figure S2: Primary sequence alignment of RyR2, between the position 3584 and 3643 (boxed) from 5 different species showing conserved (bottom stars) and non-conserved residues (bottom dots). The conserved aspartic acid at position 3638 is boxed; Figure S3: (**A**) Morphology of CPVT-hiPSC colony on mitotically-inactivated mouse embryonic fibroblasts. (**B**) Alkaline phosphatase staining of CPVT-hiPSC colonies. (**C**) Immunostaining for pluripotency markers OCT4, NANOG, and SSEA4. Nuclei were stained with DAPI. (**D**) Sequencing of *RYR2* gene showing RYR2-D3638A heterozygous point mutation in the CPVT-hiPSC. (**E**) Karyotype analysis of CPVT-hiPSC. (**F**) Semiquantitative RT-PCR of one-month-old CPVT-EB and EB of two control lines (hFiPS1.4—hiPSC line; CCTL12—hESC line) showing expression of markers of the three germ layers: endodermal markers (*AFP*, *GATA6*), mesodermal markers (*GATA4*, *VIM*), and ectodermal markers (*NOG*, *PAX6*). Glyceraldehyde-3-phosphate dehydrogenase (*GAPDH*) was used as housekeeping gene; Figure S4: (**A**) Immunostaining for cardiac troponin T (cTnT), phosphorylated cardiac troponin I (p-cTnI), and alpha-actinin (α-actinin) in CM derived from control (left) and CPVT C2.2 (right) hiPSC lines. Nuclei were stained with DAPI. Scale bar: 20 µm. (**B**) Semiquantitative RT-PCR showing expression of ryanodine receptor 2 (*RYR2*), myosin light chain 2 (*MYL2*), myosin light chain 7 (*MYL7*), alpha-cardiac actin (*ACTC1*), and connexin 43 (*GJA1*) in beating EBs derived from CPVT and control hiPSC lines, and in corresponding non-differentiated stem cells. Glyceraldehyde-3-phosphate dehydrogenase (*GAPDH*) was used as housekeeping gene. (**C**) Quantitative RT-PCR showing expression of myosin heavy chain 6 (*MYH6*, *n* = 3), myosin heavy chain 7 (*MYH7*, *n* = 3), and Ca^2+^ cycling genes: *RYR2* (*n* = 3), Calstabin2 (*FKBP1B*, *n* = 3), sarcoplasmic/endoplasmic reticulum calcium ATPase 2 (*ATP2A2*, *n* = 3), phospholamban (*PLN*, *n* = 3), and calsequestrin 2 (*CASQ2*, *n* = 3) in one-month-old beating EBs derived from CPVT and control hiPSC lines. Expression levels were normalized to *GAPDH* expression; Figure S5: (**A**) Ratio of beat rates under 1 µM ISO to beat rates at rest for hESC-, HC- and CPVT-EBs (C2.2). (**B**) Ratio of contraction forces under 1 µM ISO to contraction forces at rest for hESC-, HC- and CPVT-EBs. The dotted line indicates the ratio threshold. The number of experiments varies from 6 to 22 for each scatter plot; Figure S6: (**A**) Display of original line-scan images of Ca^2+^ transients and corresponding tracings of healthy control hiPSC-CMs (HC) and CPVT hiPSC-CMs (C2.2, CPVT) loaded with Fluo-4 at basal state. (**B**) Maximal Ca^2+^-transient amplitude in HC (white dots) and CPVT hiPSC-CMs (black dots) at rest. (**C**,**D**) Frequency of occurrence of aberrant Ca^2+^-transients (C) and diastolic SR leaky events (D) in HC and CPVT hiPSC-CMs. (**E**) Rate of RyR2 Ca^2+^ release (dF/dt_max_) in HC and CPVT hiPSC-CMs. (**F**) Area under the curve (peak area) in HC and CPVT hiPSC-CMs. The number of experiments varies from 30 to 46 for each scatter plot; Figure S7: (**A**) Display of original line-scan tracings of Ca^2+^ transients of healthy control hiPSC-CMs (HC) and CPVT hiPSC-CMs (C1.1, CPVT) under stress when 10 mM caffeine was applied shown by the black arrow. (**B**) Fractional Ca^2+^ release as a ratio between the Ca^2+^ transient amplitude prior 10 mM caffeine application and the amplitude of the following caffeine-induced Ca^2+^ transient in HC (white dots, *n* = 12) and CPVT hiPSC-CMs (C1.1, black dots, *n* = 5) under stress conditions. (**C**) Time needed to reach the maximal Ca^2+^-transient amplitude induced by 10 mM caffeine in HC (white dots, *n* = 12) and CPVT hiPSC-CMs (C1.1, black dots, *n* = 5) under stress conditions. (**D**) Quantitative RT-PCR showing expression of β_1_- (*ADRB1*, *n* = 3) and β_2_-adrenergic receptor (*ADRB2*, *n* = 3) genes in HC- and CPVT-EBs (C2.2). Expression levels were normalized to *GAPDH* expression; Figure S8: Aberrant SR Ca^2+^ handling in CPVT is not prevented by standard β-adrenergic receptor blockade. (**A**) Display of original line-scan images of Ca^2+^ transients and corresponding tracings of CPVT (C1.1) hiPSC-CMs after application of ± 5 µM METO followed by 1 µM ISO. Aberrant Ca^2+^ release events are shown by the arrows. (**B**) Maximal Ca^2+^-transient amplitude under ISO (white dots) and METO + ISO (black dots). (**C**,**D**) Frequency of occurrence of aberrant Ca^2+^-transients (**C**) and diastolic SR leaky events (**D**) under ISO and METO + ISO. (**E**) Rate of RyR2 Ca^2+^ release under ISO and METO + ISO. (**F**) Area under the curve or peak area under ISO and METO + ISO. The number of experiments varies from 23 to 30 for each scatter plot; Figure S9: AP recorded form HC and CPVT mostly indicate ventricular-like hiPSC-CM. (**A**) Repartition of cells analyzed and rejected based on their APD_90_ at 1Hz stimulation. (**B**) Distribution frequency showing the APD_90_/APD_50_ ratio for both HC (*n* = 26) and CPVT (*n* = 26) hiPSC-CMs denoting the presence of the typical plateau phase of ventricular-like APs. Scatter plot is shown in inset; Figure S10: AP recorded from HC and CPVT under 1 Hz stimulation. Examples of raw traces from current clamp experiments on HC (left) and CPVT (right) hiPSC-CM; Figure S11: AP parameters form HC and CPVT under 1Hz stimulation. (**A**) Examples of raw traces from current clamp experiments on HC (left) and CPVT (right) hiPSC-CM. AP characteristics are studied and were compared between HC (*n* = 26) and CPVT (*n* = 26) (**B**–**G**). For each comparison, the frequency distribution is shown with a scatter plot (with mean ± SEM) in inset to provide an accurate view of population distribution. The maximum depolarization speed (maximum dV/dt) (**B**), AP amplitude (**C**), overshoot (**D**), APD_20_ (**E**), APD_50_ (**F**) and APD_90_ (**G**) were studied; Figure S12: AP parameters form HC and CPVT under 2Hz stimulation. (**A**–**F**) AP characteristics are studied and were compared between HC (*n* = 25) and CPVT (*n* = 20). For each comparison, the frequency distribution is shown with a scatter plot (with mean ± SEM) in inset to provide an accurate view of population distribution. The maximum depolarization speed (maximum dV/dt) (**A**), AP amplitude (**B**), overshoot (**C**), APD_20_ (**D**), APD_50_ (**E**) and APD_90_ (**F**) were studied; Figure S13: AP parameters variations form HC and CPVT under 1 and 2 Hz stimulations. (**A**) Histogram showing the variations in AP parameters (APD_90_, APD_50_, APD_20_, overshoot, amplitude and dV/dt max) between HC and CPVT (HC as reference). Black bars indicate variations between HC and CPVT under 1Hz stimulation. Striated bars indicate variations between HC and CPVT under 2 Hz stimulation. (**B**) Histogram showing the variations in AP parameters between 1 Hz and 2 Hz (1 Hz as reference). White bars indicate the variation between 1 Hz and 2 Hz stimulations for the HC. Black bars indicate the variation between 1 Hz and 2 Hz stimulations for the CPVT.
