# Supplementary material for: Post-Translational Modifications and Diastolic Calcium Leak Associated to the Novel RyR2-D3638A Mutation Lead to CPVT in Patient-Specific hiPSC-Derived Cardiomyocytes
Source: J Clin Med. 2018 Nov 8;7(11):423. doi: 10.3390/jcm7110423 (PMC6262462; doi:10.3390/jcm7110423)
Supplement: Supplementary file 1 [file jcm-07-00423-s001.pdf]

ONLINE DATA SUPPLEMENT
Abbreviations and acronyms:

CPVT – catecholaminergic polymorphic ventricular tachycardia

HC – healthy control

MEF – mouse embryonic fibroblast

hESC - human embryonic stem cell

hiPSC - human induced pluripotent stem cell

hiPSC-CM - human induced pluripotent stem cell derived cardiomyocyte

EB - embryoid body

CM - cardiomyocyte

Ca^{2+} - calcium

RyR - ryanodine receptor

RT – reverse transcription

WT - wild type

SR – sarcoplasmic reticulum

ECC - excitation-contraction coupling

PKA – protein kinase A

CaM - calmodulin

ISO - isoproterenol

METO – metoprolol

FLEC - flecainide

Tables:

Table S1: spontaneous AP parameters

		MDP (mV)	Frequency (Hz)	dV/dt (mV/ms)	Amplitude (mV)	$APD_{90\ corrected}$ (ms)
HC	-ISO (n=26)	-66.4 ± 1.4	0.40 ± 0.04	9.2 ± 1.3	104.4 ± 3.2	301.5 ± 26.4
	+ISO (n=25)	-66.2 ± 1.2	0.41 ± 0.06	6.0 ± 1.0	102.6 ± 2.1	264.2 ± 26.3
CPVT	-ISO (n=41)	-65.6 ±1.1	0.61 ± 0.04***	8.6 ± 0.9	107 ± 2.1	376.1 ± 14.7**
	+ISO (n=20)	-61 ± 1.8$^{\#}$	0.45 ± 0.05$^{\#}$	5.7 ± 0.8$^{\#}$	97.9 ± 3.7$^{\#}$	314.7 ± 23.8$^{\#}$

dV/dt: maximum depolarization speed. MDP: maximum depolarization speed. $APD_{90\ corrected}$: AP duration at 90% of repolarization corrected using the Bazett's formula. Stars indicate differences between HC and CPVT without ISO. # indicate differences between CPVT with and without ISO. No differences were found between HC with and without ISO.

Table S2: AP parameters at different pacing

		dV/dt (mV/ms)	Amplitude (mV)	Overshoot (mV)	APD_{20} (ms)	APD_{50} (ms)	APD_{90} (ms)
1 Hz	HC (n=26)	78.9 ± 9.2	118.7 ± 2.2	40.3 ± 2.0	103 ± 15.7	183.5 ± 19.5	287 ± 20.2
	CPVT (n=26)	91.9 ± 7.1	124.5 ± 1.3*	45.6 ± 1.2	153.2 ± 19.3	293.8 ± 26.8**	417.8 ± 28.9***
2 Hz	HC (n=25)	61.2 ± 8.9	110.6 ± 2.9	31.7 ± 2.6	51.3 ± 6.1	106.4 ± 9.0	206.2 ± 13.7
	CPVT (n=20)	78.4 ± 7.5	116 ± 2.8	37.2 ± 2.5	91.4 ± 8.3***	187.1 ± 8.6****	301.4 ± 9.1****

dV/dt: maximum depolarization speed. Stars indicate differences between respective HC and CPVT.

Table S3: Paced AP parameters variations

	HC *vs.* CPVT (1Hz)	HC *vs.* CPVT (2Hz)	1 *vs.* 2Hz (HC)	1 *vs.* 2Hz (CPVT)
**dV/dt (mV/ms)**	16.5	28	-22.4	-14.7
**Amplitude (mV)**	4.9	4.9	-6.8	-6.8
**Overshoot (mV)**	13.1	17.2	-21.3	-18.5
**APD_{20} (ms)**	48.7	78.3	-50.2	-40.4
**APD_{50} (ms)**	60.1	75.8	-42	-36.3
**APD_{90} (ms)**	45.6	46.2	-28.2	-27.9

dV/dt: maximum depolarization speed. Increase in variations between HC *vs.* CPVT (1Hz) and HC *vs.* CPVT (2Hz) are highlighted in red. Decrease in variations between 1 *vs.* 2Hz (HC) and 1 *vs.* 2Hz (CPVT) are highlighted in green.

Table S4. List of primer sequences used for PCR analyses of cardiac markers.

Gene	Forward primer (5'-3')	Reverse primer (5'-3')
**Semiquantitative RT-PCR**		

RYR2	CTGGTGAGGAAGAAGCCAAG	TGTCTTCCTGGCTGTGAGTG
MYL2	TACGTTCGGGAAATGCTGAC	TTCTCCGTGGGTGATGATG
MYL7	CCGTCTTCCTCACGCTCTT	TGAACTCATCCTTGTTCACCAC
ACTC1	GCCCTGGATTTTGAGAATGA	ATGCCAGCAGATTCCATACC
GJA1	AAGTACCAAACAGCAGCGGAG	ACGAAAGGCAGACTGCTCATC
GAPDH	GGAAGGTGAAGGTCGGAGTCA	ATGGGTGGAATCATATTGGAACA
**Quantitative RT-PCR**		
MYH6	CTCCTACGCAACTGCCGATA	GGGATGATGCAACGCAC
MYH7	GAGAAACACGCAACAGAGAAC	GACCTTGTCCTCCTCGG
RYR2	GCTCTACTCAGCCGTCT	TGGATGTCAATCAGCAGGTC
FKBP1B	CGGAGACGGAAGGACAT	GGCTGCACCCTCTTCAA
ATP2A2	CTTGGCTATTGGCTGTTACG	TTGCACAATCCACGCCT
PLN	TAAACACCCGTAAGACTTCATAC AAC	ATAGCTGAGCGAGTGAGG
CASQ2	CCGAGTGGTAAGTCTTTCCG	TTATGTTCAAGGACCTGGGC
GAPDH	ACATCATCCCTGCCTCTAC	CCTGCTTCACCACCTTCTT
ADRB1	TCGTGTGCACCGTGTGGGCC	AGGAAACGGCGCTCGCAGCTGTCG
ADRB2	GCCTGCTGACCAAGAATAAGGCC	CCCATCCTGCTCCACCT

Table S5. Primary and secondary antibodies used.

**Marker**	**Primary antibody**	**Dilution**	**Company**	**Cat. No.**	**Secondary antibody**	**Dilution**	**Company**	**Cat. No.**
NANOG	Rabbit polyclonal	1:200	Santa Cruz	sc-33759	Alexa Fluor 594 donkey anti-rabbit	1:500	Invitrogen	A-21207
OCT4	Mouse monoclonal	1:200	Santa Cruz	sc-5279	Alexa Fluor 594 donkey anti-mouse	1:500	Invitrogen	A-21203

SSEA4	Mouse monoclonal	1:100	Millipore	MAB4304	Alexa Fluor 594 goat anti-mouse	1:500	Invitrogen	A-11005
cTnT	Goat polyclonal	1:250	Santa Cruz	sc-8121	Alexa Fluor 488 donkey anti-goat	1:500	Invitrogen	A-11055
p-cTnI	Rabbit polyclonal	1:500	Cell Signaling	4004S	Alexa Fluor 594 donkey anti-rabbit	1:500	Invitrogen	A-21207
RyR	Mouse monoclonal	1:1000	Abcam	2868	IRDye 800CW goat anti-mouse	1:30000	Li-Cor	926-32210
FKBP12.6	Goat polyclonal	1:500	R&D	AF4174-SP	IRDye 800CW donkey anti-goat	1:30000	Li-Cor	925-32214
Spinophilin	Goat monoclonal	1:1000	Santa Cruz	sc-373974	IRDye 800CW donkey anti-goat	1:30000	Li-Cor	925-32214
PP2A-alpha	Mouse monoclonal	1:1000	BDBiosciences	610555	IRDye 800CW goat anti-mouse	1:30000	Li-Cor	926-32210
α-actinin	Mouse monoclonal	1:1000	Sigma-Aldrich	A7811	Alexa Fluor 488 goat anti-mouse	1:500	Invitrogen	A-11001

Table S6. Used drugs.

Drug	Company	Cat. No.	Final concentration	Buffer
Isoproterenol	Sigma-Aldrich	1351005	1µM	Tyrode's solution
S107	Merck	500469	5µM	Tyrode's solution
Metoprolol	Sigma-Aldrich	BP540	5µM	Tyrode's solution
Flecainide	Sigma-Aldrich	F6777	5µM	Tyrode's solution

Materials and methods

Protein sequence analysis and molecular modelling of the RyR2-D3638A mutant

RyR2 protein sequence alignment between the position 3584 and 3643 was performed using Uniprot (www.uniprot.org/align). A total of 5 different species were compared. In order to understand how D3638A substitution may change the structure of human RyR2, we modelled this mutant based on the known structure of the pig RyR2 (PDB code 5GO9)[1] by using Homology and Discovery modules of InsightII program [2].

CPVT-hiPSC generation and maintenance of pluripotent stem cells

All subjects gave their informed consent for inclusion before they participated in the study. The study was conducted in accordance with the Declaration of Helsinki, and the protocol (10-02214) was approved by the Ethics Committee of the UCSF medical center (CA, USA). The patient-specific hiPSC clones were generated by reprogramming of primary skin fibroblasts using Human STEMCCA Constitutive Polycistronic (OKSM) Lentivirus Reprogramming Kit (Millipore, SCP544) according to the manufacturer's protocol. Briefly, on day-0, fibroblasts were seeded at density of $6x10^4$ cells per 40 mm diameter Petri dish (TPP, 93040) in the MEF medium consisting of Knockout Dulbecco's modified Eagle's medium (KO-DMEM; Gibco), 10% heat-inactivated fetal bovine serum (Invitrogen), 1% L-glutamine (Gibco), 1% non-

essential amino acids (PAA), 0.5% penicillin-streptomycin (Gibco), and 0.1 mM β-mercaptoethanol (Sigma-Aldrich). On day-1, cells were transduced in the presence of 5 µg/ml polybrene with the volume of virus to achieve an MOI (multiplicity of infection) of 75. In the same way, the second viral infection was done next day. From day 3 MEF medium was changed daily till day-6 when the virus-infected cells were treated with Accumax (Millipore, SCR006) for 9 min at 37°C and plated at density of $2x10^4$ and $3x10^4$ per well of 6-well plate (TPP,) containing inactivated mouse embryonic fibroblasts (MEFs, $3x10^5$ cells per well of 6-well plate) in the HES medium consisting of DMEM/F-12 (Gibco), 15% KnockOut Serum Replacement (KO-SR; Gibco), 1% L-glutamine, 1% non-essential amino acids, 0.5% penicillin-streptomycin, and 0.1 mM β-mercaptoethanol, supplemented with 10 ng/ml FGF2 (R&D, 233-FB), 1X TGF-β RI Kinase Inhibitor VI Supplement, and 1X HDAC Inhibitor-A Supplement, both from the Human iPS Cell Boost Supplement Kit (Millipore, SCM088). The HES medium with supplements was changed every second day. $4x10^5$ MEFs per well of 6-well plate were added on day-12 and every-second-day changing of HES medium with supplements was continued till day-18 when first hiPSC-like colonies were picked. Till day-25 more colonies were selected for passage and finally three CPVT-hiPSC clones were established: C1.1, C2.2, and C2.5.

Generated CPVT-hiPSC clones as well as pluripotent stem cell lines that were used as healthy controls in this study, UEFhfiPS1.4 [3] and CCTL12 previously published [4,5] were maintained as colonies on mitotically inactivated MEF feeder in HES medium with 10 ng/ml human FGF2. Cells were manually dissected and passaged every 4-6 days.

Karyotype analysis

For cytogenetic analysis hiPSC metaphase arrest was induced using 0.1 µg/ml Colcemid (Biochrom) for 2 h at 37°C. After trypsinization, cells were incubated in pre-warmed at 37°C

hypotonic 75 mM KCl solution, for 20 min at 37°C. Prefixation was done by addition of few drops of ice-cold fixative solution (methanol: glacial acetic acid = 3 : 1 (v/v)) and was kept for 4 min on ice. After centrifugation at 1000 rpm for 3 min at room temperature, pellet was resuspended in ice-cold fixative solution and this step was repeated for 3 times. After the second addition of fixative solution, it was kept for 1 h at -20°C. Chromosomal G-banding analysis was performed in the department of human cytogenetics at the CHU Arnaud de Villeneuve (Montpellier, France).

Genomic sequencing

In order to confirm the presence of point mutation p.D3638A in CPVT-hiPSCs, genomic DNA was isolated using phenol : chloroform : isoamyl alcohol (Ph:Ch:IAA = 25:24:1, v/v/v) extraction. Briefly, cells were resuspended in Ph:Ch:IAA mixture. After centrifugation, collected upper phase was mixed with the equal volume of Ch:IAA (24:1, v/v), and this step was repeated for 3 times. To the final collected volume (V_f) was added V_f / 10 of 3 M sodium acetate, pH 5.2, and 2.5 x V_f of ice-cold 100% ethanol, and it was incubated overnight at 4°C. Next day precipitated DNA was washed with 70% ethanol and resuspended in TE buffer (10 mM Tris, 1 mM EDTA, pH 8). The relevant fragment of the *RYR2* gene was amplified by PCR using the primers: F-5’-TACCTTTCAGGCATCGGG-3' and R-5‘-TAATGACTGCTCACGCCAAC-3', resulting in the amplicon length of 182 bp. DNA was purified using QIAquick PCR Purification Kit (Qiagen) and sample was sent for sequencing.

Alkaline phosphatase activity

Alkaline phosphatase activity was determined in hiPSCs that were fixed with 4% paraformaldehyde (PFA) for 4 min at room temperature, and after washing with 1x phosphate-

buffered saline (PBS), using Alkaline Phosphatase Blue Microwell Substrate kit (Sigma-Aldrich).

RT-PCR and qRT-PCR

One-month-old beating EBs and undifferentiated hiPSC colonies were used for RNA extraction. Total RNA was isolated using RNA blue (Top-Bio, cat. no. R012) followed by cDNA synthesis using Revert Aid H Minus First Strand cDNA Synthesis Kit (Fermentas, # K1632) for semiquantitative RT-PCRs, and Verso cDNA Synthesis Kit (Thermo Scientific, # AB-1453/A) for qRT-PCR, according to manufacturers' protocols. For semi quantitative RT-PCR for detection of markers of 3 germ layers cDNA was amplified using One Taq DNA Polymerase (New England Biolabs, M0480G), while used primers have already been described [6]. For detection of cardiac markers cDNA was amplified by Taq DNA Polymerase (Fermentas, # EP0402) in the case of semiquantitative RT-PCR, and LightCycler 480 SYBR Green I Master (Roche, 04707516001) for qRT-PCR. Glyceraldehyde-3-Phosphate Dehydrogenase (*GAPDH*) was used as a reference gene. Primers for detection of cardiac markers are listed in Table S4.

Immunocytochemistry

For detection of pluripotency markers NANOG, OCT4, and SSEA4, undifferentiated hiPSC colonies on mitotically inactivated MEFs were fixed with 4% PFA for 1 h on ice, washed with 1x PBS, permeabilized with 0.25% Triton in 1x PBS for 10 min at room temperature (for detection of nuclear markers NANOG and OCT4), and blocked with 1% bovine serum albumin (BSA) in 1x PBS for 30 min at room temperature (for all three markers). Incubation with primary antibodies diluted in 1% BSA in 1x PBS was at 4°C, overnight, and next day with secondary antibody for 1 h at room temperature. After that samples were mounted in Moviol-

4,6-diamidino-2-phenylindole (DAPI; Sigma-Aldrich). The list of used primary and secondary antibodies is given in Table S5.

Cardiac differentiation

HC- and CPVT-hiPSCs as well as hESCs were differentiated into CMs using both, the 2D (monolayer) protocol and 3D (embryoid body (EB)-based) protocol as previously published [4,7].

Embryoid body dissociation and immunocytochemistry

Prior to the staining of cardiac markers, beating (contracting) EBs were dissociated in order to get isolated CMs by collecting and washing EBs two times in Ca^{2+}-free solution (120 mM NaCl, 5.4 mM KCl, 5 mM $MgSO_4$, 5 mM sodium pyruvate, 20 mM glucose, 20 mM taurine, and 10 mM HEPES; pH 6.9). EBs were spin down at 15g for 3 min, Ca^{2+}-free solution was exchanged for the second time, and EBs were left for 20 min at room temperature before centrifugation. After spinning down and removing the supernatant, EBs were incubated for 3-5 min at 37°C in digestion solution (Ca^{2+}-free solution supplemented with 0.8 mg/ml type II collagenase from *Clostridium histolyticum* (Sigma-Aldrich) and 0.04 mg/ml type XIV protease from *Streptomyces griseus* (Sigma-Aldrich) pre-warmed to 37°C, with periodic shaking. After spinning down at 170g for 3 min the pellet was resuspended in 1 ml of Kraft-Bruhe (KB) solution (85 mM KCl, 30 mM K_2HPO_4, 1 mM EGTA, 2 mM ATP-Na_2, 5 mM sodium pyruvate, 5 mM creatine, 20 mM taurine, and 20 mM glucose; pH 7.3) pre-warmed to 37°C, and incubated for 20 min at 37°C, with periodic shaking. Finally, cells were plated on the gelatin-coated 12 mm diameter coverslips in the MEF medium.

Derived CMs were fixed with 4% PFA for 1 h on ice and washed with 1x PBS. Only for detection of phosphorylated cardiac troponin I (p-cTnI), before fixation CMs were treated with

1 µM isoproterenol for 2 min at 37°C. A blocking solution 1 (1% BSA, 0.05% Triton in 1x PBS) was used for 15 min at room temperature. After washing with 1x PBS, there was incubation with blocking solution 2 (1% BSA, 0.03% Tween in 1x PBS) for 1 h at room temperature. Primary antibodies were diluted in 1x PBS with 0.05% Tween, and incubation took place overnight at 4°C. Incubation with secondary antibodies and DAPI diluted in 1x PBS was for 1 h at room temperature. After washing with 1x PBS, samples were mounted in Mowiol. DAPI dilution was 1:1000. Detailed list of used primary and secondary antibodies is given in Table S5.

Atomic force microscopy (AFM)

Homogenous EBs in size and shape of 30 to 60 days-old were plated on the gelatin-coated 40 mm diameter Petri dishes (TPP) in MEF medium one or two days prior to measurements on the AFM. MEF medium was exchanged for Tyrode's solution (140 mM NaCl, 4 mM KCl, 1 mM $MgCl_2$, 5 mM HEPES, 10 mM glucose, and 1.8 mM $CaCl_2$) at least 10 min prior each measurement. For each EB was done measurement of 10 min in Tyrode's solution, followed by exchange to Tyrode's solution supplemented with 1 µM isoproterenol. To ensure drug diffusion into the EB, 10 min of incubation (so-called stabilization period) was reached before measuring the mechano-biological properties of the EB for another 10 min. To test the effect of Rycal S107, EBs were treated with 5 µM S107 (Cayman Chemical) overnight at 37°C and the next following day in the same way were done 10 min measurement series in Tyrode's solution and under β-adrenergic receptor stimulation with 1 µM isoproterenol.

JPK Nanowizard 3 (JPK) BioAFM microscope placed on Olympus IX81 (Olympus corporation) inverted optical microscope was used for measurements. Uncoated silicon nitride AFM cantilevers HYDRA2R-50N from Applied NanoStructures (Mountain View) were used for all experiments. All the probes were calibrated prior the biomechanical experiments.

Sensitivity of AFM setup was calibrated by force-distance curve measurement (setpoint value 1.5 V, lifting height 600 nm), giving typical values between 25.5 and 32.5 nm/V. Stiffness of the cantilever was subsequently calibrated by thermal noise measurement, typical values found here were lying between 0.061 and 0.090 N/m. AFM cantilever was placed over the EB surface by motorized stage allowing X-Y movement of the dish, when the situation was monitored with an optical microscope. The cantilever was afterwards introduced into the constant contact with EB surface, with following feedback loop parameters: setpoint 6.0 nN, iGain 0.01 and pGain 50. The principle of feedback loop function is to keep the cantilever-EB interaction force constant at 6 nN by force compensation, which is shown in oscilloscope window as mechanocardiogram (MCG). Recorded data sets were evaluated using in-house developed algorithm implemented in MatLab. Detailed list of used compounds and drugs is given in Table S3.

Measurement of cytosolic Ca^{2+} variation

After dissociation of 30 to 60 day-old contracting EBs, hiPSC-CMs were loaded with 3 µM Fluo-4 AM Ca^{2+} indicator (Molecular Probes) in Tyrode's solution for 20 min. Cells were placed in an experimental chamber on the stage of an inverted microscope. Ca^{2+} images were recorded with an inverted confocal microscope (Zeiss LSM 510) equipped with a 63x lens (oil immersion, numerical aperture, N.A. = 1.4). Confocal images were obtained in line scan mode (i.e. x-t mode, 1.53 ms per line; 512 pixels x 5,000 lines). The SR Ca^{2+} content was measured in intact CMs loaded with Fluo-4 and upon application of 10 mM caffeine to release the SR Ca^{2+} store in plane (frame) scan mode in x-y mode at a rate of 1 image/0.782 s. To enable comparisons between cells, changes in the Fluo-4 fluorescence signal (ΔF) were normalized by basal fluorescence (F_0). All data were extracted using AIM 4.2 (Zeiss). Maximal amplitudes and event frequencies were extracted and analyzed from raw data by in-house developed

algorithm implemented in Python (version 3.0) (https://asalykin.github.io/PeakInspector/) and Prism (version 6.0; GraphPad). For experiments with metoprolol (Sigma-Aldrich), hiPSC-CMs were loaded with 5 µM for 10 min prior the measurement of cytosolic Ca^{2+} variation. Detailed list of used compounds and drugs is given in Table S6.

Electrophysiology

Patch clamp experiments were performed using an Axopatch 200B amplifier (Axon Instruments) at room temperature at least 6 days after the dissociation of the hiPSC–CMs. The pipettes were made from borosilicate glass capillaries and were fire polished. Action potentials (APs) were evaluated using the whole-cell configuration of the patch clamp technique (in current clamp mode with a sampling frequency of 5000 Hz). Spontaneous APs were recorded using the gap free mode during which electrical activity is recorded without intervention. After artificially lowering the maximum diastolic potential to -80 mV, APs were also elicited using a 3 ms, 200 to 2500-pA rectangular current pulse injection at several frequencies. The patch pipets (resistance 2-8 mΩ) were filled with a solution containing (in mM): 10 NaCl, 122 KCl, 1 $MgCl_2$, 1 EGTA, and 10 Hepes. The pH was adjusted to 7.3 with KOH. The external current clamp solution was composed of (in mM): 154 NaCl, 5.6 KCl, 2 $CaCl_2$, 1 $MgCl_2$, 8 D-glucose, and 10 Hepes. The pH was adjusted to 7.3 with NaOH. Data were analyzed using custom-written MatLab (The MathWorks Inc.), Microsoft Excel and Prism.

Immunoprecipitation and immunoblot analyses

Differentiated HC and CPVT embryoid bodies of at least 30 days old were incubated with a lysis buffer composed of 35 mM NaF, 10 mM Tris maleate pH 6.8, 1.0 mM Na_3VO_4, 1% Triton-X100, and protease inhibitors. RyR2 channels were immunoprecipitated by incubating 75 µg of cell lysate using an anti-RyR antibody for 2 hours at 4°C in 0.5 ml of a modified RIPA

buffer (50 mM Tris-HCl pH 7.4, 0.9% NaCl, 5.0 mM NaF, 1.0 mM Na_3VO_4, 0.5% Triton-X100, and protease inhibitors). The immune samples were then incubated with protein A/G magnetic beads (Pierce) at 4 °C for 2 h, after which the beads were washed three times with RIPA buffer. Proteins were then separated using a 6-15% SDS-PAGE gradient gel, blotted onto nitrocellulose membranes (0.2 µm; GE Healthcare), and incubated overnight at 4 °C with primary antibodies: anti-RyR2, anti-FKBP12.6, anti-spinophilin and anti-PP2A. Levels of RyR2 bound proteins were normalized to the total RyR2 immunoprecipitated (arbitrary units). All immunoblots were developed using the Odyssey system (LI-COR) with IR labeled secondary antibodies for 1 h at room temperature.

Statistical analysis

Normality was tested using the Shapiro-Wilk test. An unpaired *t* test was used to compare 2 independent groups with parametric distribution. A Mann-Whitney test was performed for comparing 2 independent groups non-parametric distribution. A 2-way-analysis of variance (ANOVA) was used to compare more than 2 groups ± treatment with normal distribution followed by post-hoc test. We performed a Kruskal-Wallis test to compare more than 2 groups ± treatment with non-parametric distribution. All data are expressed as mean ± SEM. A value of $p < 0.05$ was considered significant. *, $p < 0.05$, **, $p < 0.01$ otherwise specified. Data analysis and statistics were done with Prism.

Figure Legends:

Figure S1. (**A)** An exercise treadmill test showed the disappearance of VTs when the proband was treated with 100 mg flecainide (predominantly RBBB, Inferior axis). (**B)** DNA sequence chromatogram of the proband showing the heterozygous single nucleotide substitution (indicated by M) adenine (A) to cytosine (C) of the *RyR2* gene nucleotide sequence (RYR2-688,730A>C). This mutation results in an aspartic acid to alanine substitution at the 3638th position (Asp3638Ala or D3638A) of the RyR2 protein.

Figure S2. (**A**) Primary sequence alignment of RyR2, between the position 3584 and 3643 (boxed) from 5 different species showing conserved (bottom stars) and non-conserved residues (bottom dots). The conserved aspartic acid at position 3638 is boxed.

Figure S3. (**A**) Morphology of CPVT-hiPSC colony on mitotically-inactivated mouse embryonic fibroblasts. (**B**) Alkaline phosphatase staining of CPVT-hiPSC colonies. (**C**) Immunostaining for pluripotency markers OCT4, NANOG, and SSEA4. Nuclei were stained with DAPI. (**D**) Sequencing of *RYR2* gene showing RYR2-D3638A heterozygous point mutation in the CPVT-hiPSC. (**E**) Karyotype analysis of CPVT-hiPSC. (**F**) Semiquantitative RT-PCR of one-month-old CPVT-EB and EB of two control lines (hFiPS1.4 – hiPSC line; CCTL12 – hESC line) showing expression of markers of the three germ layers: endodermal markers (*AFP*, *GATA6*), mesodermal markers (*GATA4*, *VIM*), and ectodermal markers (*NOG*, *PAX6*). Glyceraldehyde-3-phosphate dehydrogenase (*GAPDH*) was used as housekeeping gene.

Figure S4. (**A**) Immunostaining for cardiac troponin T (cTnT), phosphorylated cardiac troponin I (p-cTnI), and alpha-actinin (α-actinin) in CM derived from control (left) and CPVT

C2.2 (right) hiPSC lines. Nuclei were stained with DAPI. Scale bar: 20 µm. (**B**) Semiquantitative RT-PCR showing expression of ryanodine receptor 2 (*RYR2*), myosin light chain 2 (*MYL2*), myosin light chain 7 (*MYL7*), alpha-cardiac actin (*ACTC1*), and connexin 43 (*GJA1*) in beating EBs derived from CPVT and control hiPSC lines, and in corresponding non-differentiated stem cells. Glyceraldehyde-3-phosphate dehydrogenase (*GAPDH*) was used as housekeeping gene. (**C**) Quantitative RT-PCR showing expression of myosin heavy chain 6 (*MYH6*, n=3), myosin heavy chain 7 (*MYH7*, n=3), and Ca^{2+} cycling genes: *RYR2* (, n=3), Calstabin2 (*FKBP1B*, n=3), sarcoplasmic/endoplasmic reticulum calcium ATPase 2 (*ATP2A2*, n=3), phospholamban (*PLN*, n=3), and calsequestrin 2 (*CASQ2*, n=3) in one-month-old beating EBs derived from CPVT and control hiPSC lines. Expression levels were normalized to *GAPDH* expression.

Figure S5. (A) Ratio of beat rates under 1 µM ISO to beat rates at rest for hESC-, HC- and CPVT-EBs (C2.2). **(B)** Ratio of contraction forces under 1 µM ISO to contraction forces at rest for hESC-, HC- and CPVT-EBs. The dotted line indicates the ratio threshold. The number of experiments varies from 6 to 22 for each scatter plot.

Figure S6. (**A**) Display of original line-scan images of Ca^{2+} transients and corresponding tracings of healthy control hiPSC-CMs (HC) and CPVT hiPSC-CMs (C2.2, CPVT) loaded with Fluo-4 at basal state. (**B)** Maximal Ca^{2+}-transient amplitude in HC (white dots) and CPVT hiPSC-CMs (black dots) at rest. (**C-D)** Frequency of occurrence of aberrant Ca^{2+}-transients **(C)** and diastolic SR leaky events **(D)** in HC and CPVT hiPSC-CMs. **(E)** Rate of RyR2 Ca^{2+} release (dF/dt_{max}) in HC and CPVT hiPSC-CMs. **(F)** Area under the curve (peak area) in HC and CPVT hiPSC-CMs. The number of experiments varies from 30 to 46 for each scatter plot.

Figure S7. (**A**) Display of original line-scan tracings of Ca^{2+} transients of healthy control hiPSC-CMs (HC) and CPVT hiPSC-CMs (C1.1, CPVT) under stress when 10 mM caffeine was applied shown by the black arrow. (**B**) Fractional Ca^{2+} release as a ratio between the Ca^{2+} transient amplitude prior 10 mM caffeine application and the amplitude of the following caffeine-induced Ca^{2+} transient in HC (white dots, n=12) and CPVT hiPSC-CMs (C1.1, black dots, n=5) under stress conditions. (**C**) Time needed to reach the maximal Ca^{2+}-transient amplitude induced by 10 mM caffeine in HC (white dots, n=12) and CPVT hiPSC-CMs (C1.1, black dots, n=5) under stress conditions. **(D)** Quantitative RT-PCR showing expression of β_1-(*ADRB1*, n=3) and β_2-adrenergic receptor (*ADRB2*, n=3) genes in HC- and CPVT-EBs (C2.2). Expression levels were normalized to *GAPDH* expression.

Figure S8. Aberrant SR Ca^{2+} handling in CPVT is not prevented by standard β-adrenergic receptor blockade. (A) Display of original line-scan images of Ca^{2+} transients and corresponding tracings of CPVT (C1.1) hiPSC-CMs after application of ± 5 µM METO followed by 1 µM ISO. Aberrant Ca^{2+} release events are shown by the arrows. (**B)** Maximal Ca^{2+}-transient amplitude under ISO (white dots) and METO + ISO (black dots). **(C-D)** Frequency of occurrence of aberrant Ca^{2+}-transients **(C)** and diastolic SR leaky events **(D)** under ISO and METO + ISO. (**E)** Rate of RyR2 Ca^{2+} release under ISO and METO + ISO. **(F)** Area under the curve or peak area under ISO and METO + ISO. The number of experiments varies from 23 to 30 for each scatter plot.

Figure S9. AP recorded form HC and CPVT mostly indicate ventricular-like hiPSC-CM. (A) Repartition of cells analyzed and rejected based on their APD_{90} at 1Hz stimulation. **(B)** Distribution frequency showing the APD_{90}/APD_{50} ratio for both HC (n=26) and CPVT (n=26)

hiPSC-CMs denoting the presence of the typical plateau phase of ventricular-like APs. Scatter plot is shown in inset.

Figure S10. AP recorded from HC and CPVT under 1Hz stimulation. Examples of raw traces from current clamp experiments on HC (left) and CPVT (right) hiPSC-CM.

Figure S11. AP parameters form HC and CPVT under 1Hz stimulation. (A) Examples of raw traces from current clamp experiments on HC (left) and CPVT (right) hiPSC-CM. AP characteristics are studied and were compared between HC (n=26) and CPVT (n=26) (**B-G**). For each comparison, the frequency distribution is shown with a scatter plot (with mean ± SEM) in inset to provide an accurate view of population distribution. The maximum depolarization speed (maximum dV/dt) (**B**), AP amplitude (**C**), overshoot (**D**), APD_{20} (**E**), APD_{50} (**F**) and APD_{90} (**G**) were studied.

Figure S12. AP parameters form HC and CPVT under 2Hz stimulation. (A-F) AP characteristics are studied and were compared between HC (n=25) and CPVT (n=20). For each comparison, the frequency distribution is shown with a scatter plot (with mean ± SEM) in inset to provide an accurate view of population distribution. The maximum depolarization speed (maximum dV/dt) (**A**), AP amplitude (**B**), overshoot (**C**), APD_{20} (**D**), APD_{50} (**E**) and APD_{90} (**F**) were studied.

Figure S13. AP parameters variations form HC and CPVT under 1 and 2Hz stimulations. (A) Histogram showing the variations in AP parameters (APD_{90}, APD_{50}, APD_{20}, overshoot, amplitude and dV/dt max) between HC and CPVT (HC as reference). Black bars indicate variations between HC and CPVT under 1Hz stimulation. Striated bars indicate variations

between HC and CPVT under 2Hz stimulation. (**B**) Histogram showing the variations in AP parameters between 1Hz and 2Hz (1Hz as reference). White bars indicate the variation between 1Hz and 2Hz stimulations for the HC. Black bars indicate the variation between 1Hz and 2Hz stimulations for the CPVT.

Figures

Figure S1

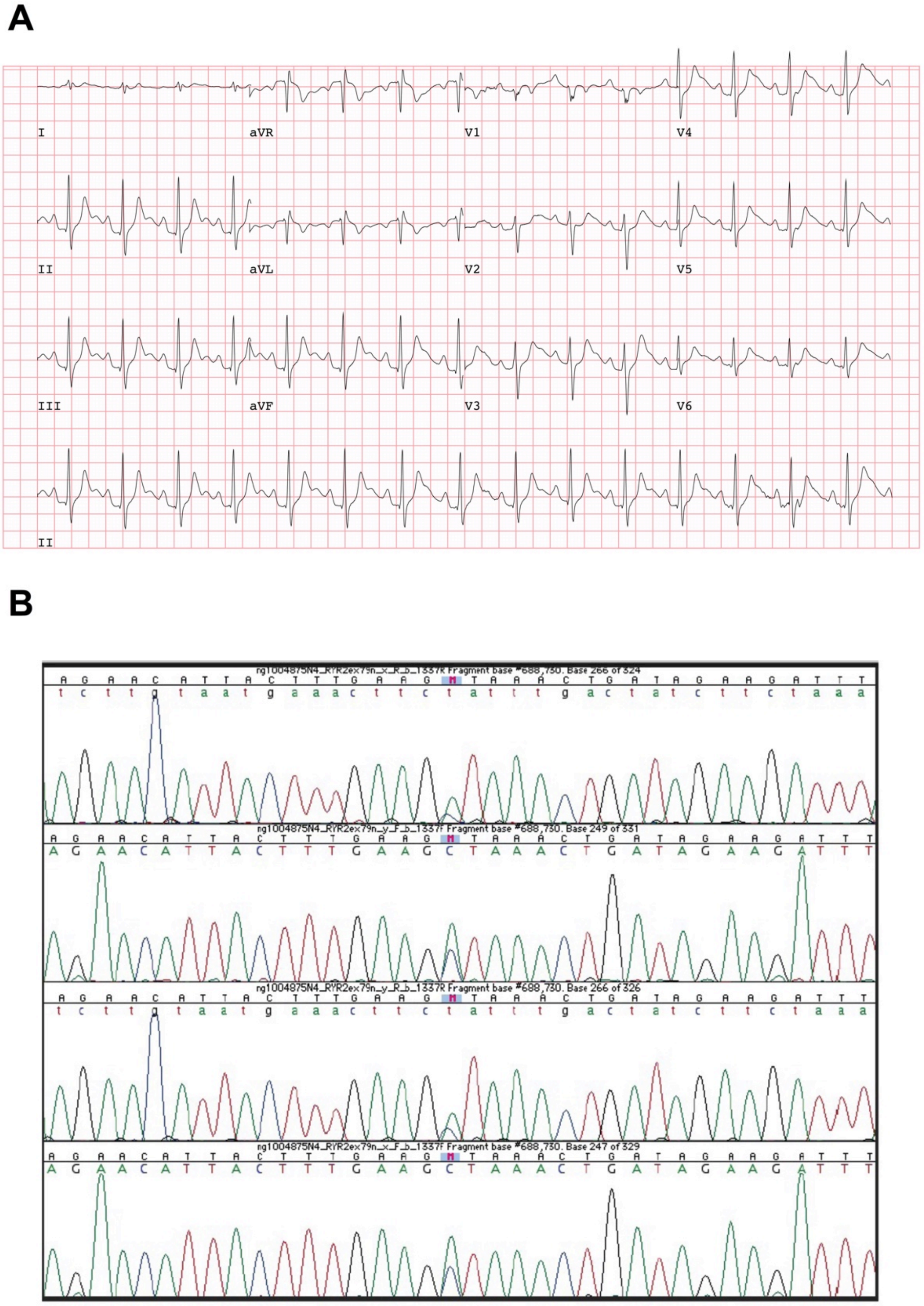

Figure S2

A

```
Q92736 RYR2_HUMAN     3524 IRWQMALYKDLPNRTDDTSDPEKTVERVLDIANVLFHLEQKSKRVGRRHYCLVEHPQRSK   3583
E9Q401 RYR2_MOUSE     3523 IRWQMALYKDLPNRTEDPSDPERTVERVLGIANVLFHLEQKSKYTGRGYFSLVEHPQRSK   3582
B0LPN4 RYR2_RAT       3514 IRWQMALYKDLPNRAEDTSDPERTVERVLDIANVLFHLEQ------------VEHPQRSK   3561
H0VB77 H0VB77_CAVPO   3468 IRWQMALYKDLPNRTEDTSDPERTVERVLDIANVLFHLEQKSKCIGRRYYNLVEHPQRSK   3527
F1PEE8 F1PEE8_CANFA   3509 IRWQMALYKDLPNRTEDTSDPEKTVERVLDIANVLFHLEQVRFCIPFIILPQVEHPQRSK   3568
                            **************::* ****:****** **********            ********

Q92736 RYR2_HUMAN     3584 KAVWHKLLSKQRKRAVVACFRMAPLYNLPRHRAVNLFLQGYEKSWIETEEHYFEDKLIED   3643
E9Q401 RYR2_MOUSE     3583 KAVWHKLLSKQRKRAVVACFRMAPLYNLPRHRAVNLFLQGYEKSWIETEEHYFEDKLIED   3642
B0LPN4 RYR2_RAT       3562 KAVWHKLLSKQRKRAVVACFRMAPLYNLPRHRAVNLFLQGYEKSWIETEEHYFEDKLIED   3621
H0VB77 H0VB77_CAVPO   3528 KAVWHKLLSKQRKRAVVACFRMAPLYNLPRHRAVNLFLQGYEKSWIETEEHYFEDKLIED   3587
F1PEE8 F1PEE8_CANFA   3569 KAVWHKLLSKQRKRAVVACFRMAPLYNLPRHRAVNLFLQGYEKSWIETEEHYFEDKLIED   3628
                            ************************************************************

Q92736 RYR2_HUMAN     3644 LAKPGAEPPEEDEGTKRVDPLHQLILLFSRTALTEKCKLEEDFLYMAYADIMAKSCHDEE   3703
E9Q401 RYR2_MOUSE     3643 LAKPGAELPEEDEAMKRVDPLHQLILLFSRTALTEKCKLEEDFLYMAYADIMAKSCHDEE   3702
B0LPN4 RYR2_RAT       3622 LAKPGSELPEEDEAMKRVDPLHQLILLFSRTALTEKCKLEEDFLYMAYADIMAKSCHDEE   3681
H0VB77 H0VB77_CAVPO   3588 LAKPGAKPPEEDEAAKRVDPLHQLILLFSRTALTEKCKLEEDFLYMAYADIMAKSCHDEE   3647
F1PEE8 F1PEE8_CANFA   3629 LAKPGADPPEEEESTKRVDPLHQLILLFSRTALTEKCKLEEDFLYMAYADIMAKSCHDEE   3688
                            *****:. ***:*. *********************************************
```

Figure S3

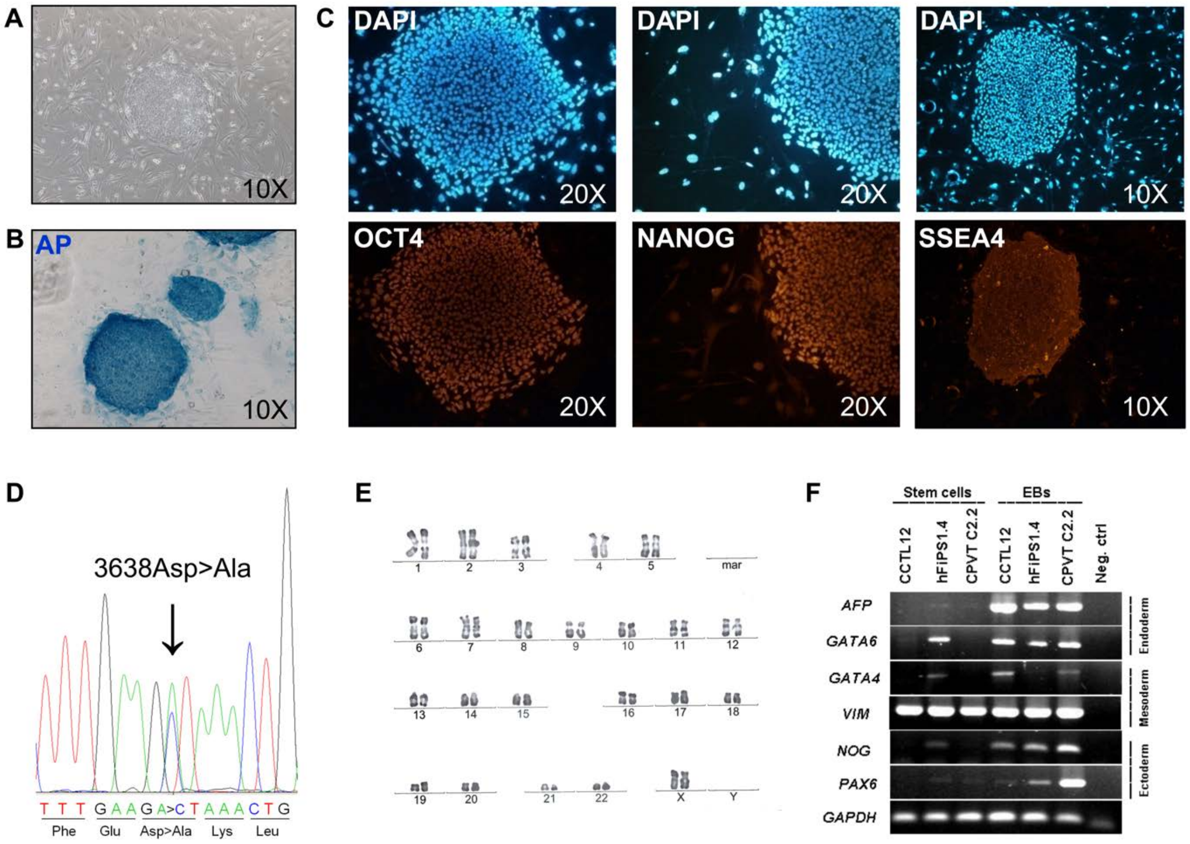
A
10X
B
AP
10X
C
DAPI
20X
DAPI
20X
DAPI
10X
OCT4
20X
NANOG
20X
SSEA4
10X
D
3638Asp>Ala
T T T G A A G A>C T A A A C T G
Phe Glu Asp>Ala Lys Leu
E
1 2 3 4 5 mar
6 7 8 9 10 11 12
13 14 15 16 17 18
19 20 21 22 X Y
F
Stem cells
EBs
CCTL12
hFiPS1.4
CPVT C2.2
CCTL12
hFiPS1.4
CPVT C2.2
Neg. ctrl
AFP
GATA6
GATA4
VIM
NOG
PAX6
GAPDH
Endoderm
Mesoderm
Ectoderm

Figure S4

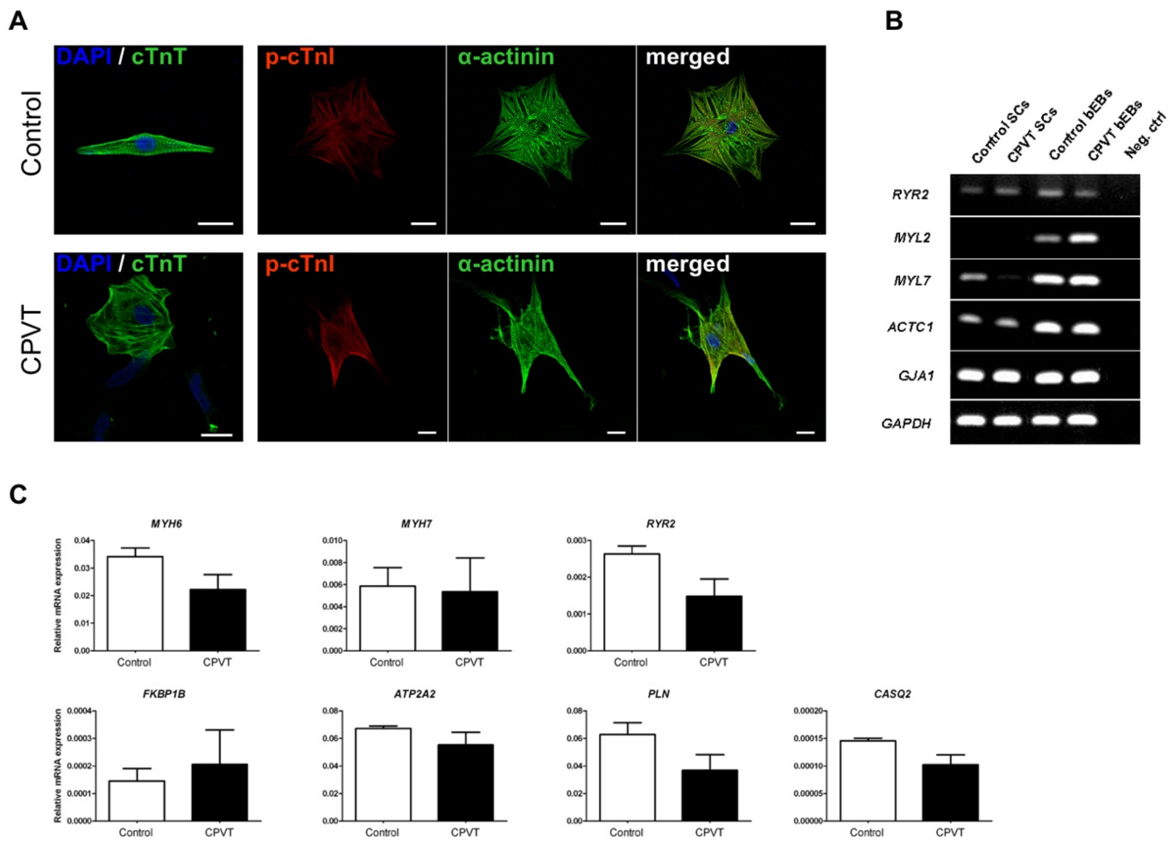
A
B
C
DAPI / cTnT
p-cTnI
α-actinin
merged
Control
CPVT
Control SCs
CPVT SCs
Control bEBs
CPVT bEBs
Neg. ctrl
RYR2
MYL2
MYL7
ACTC1
GJA1
GAPDH
MYH6
MYH7
RYR2
FKBP1B
ATP2A2
PLN
CASQ2
Relative mRNA expression
Control
CPVT

Figure S5

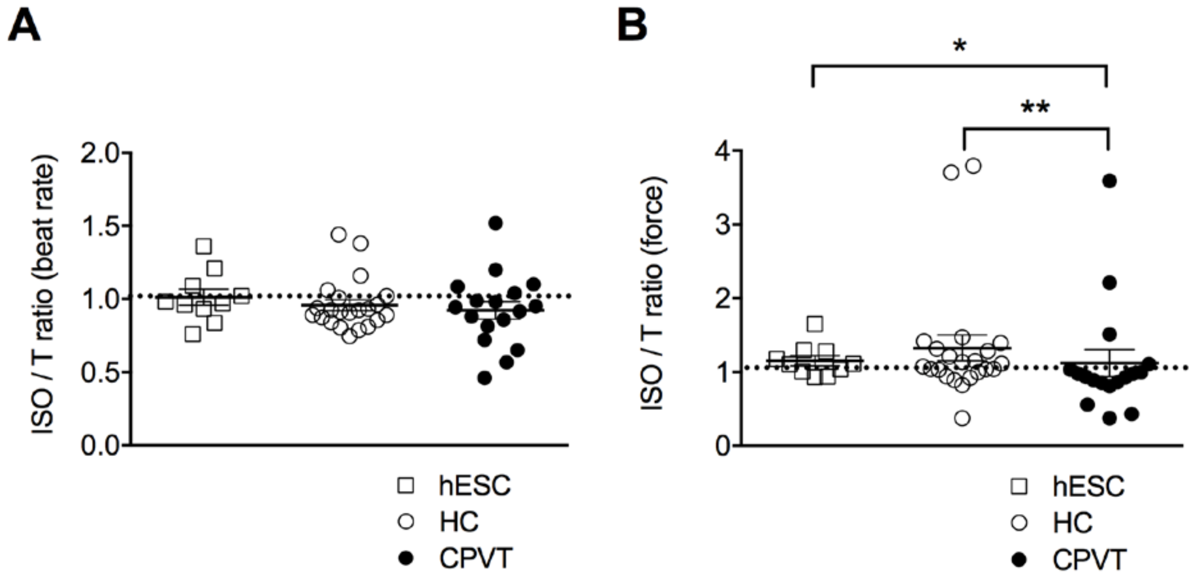
A
ISO / T ratio (beat rate)
2.0
1.5
1.0
0.5
0.0
hESC
HC
CPVT
B
*
**
ISO / T ratio (force)
4
3
2
1
0
hESC
HC
CPVT

Figure S6

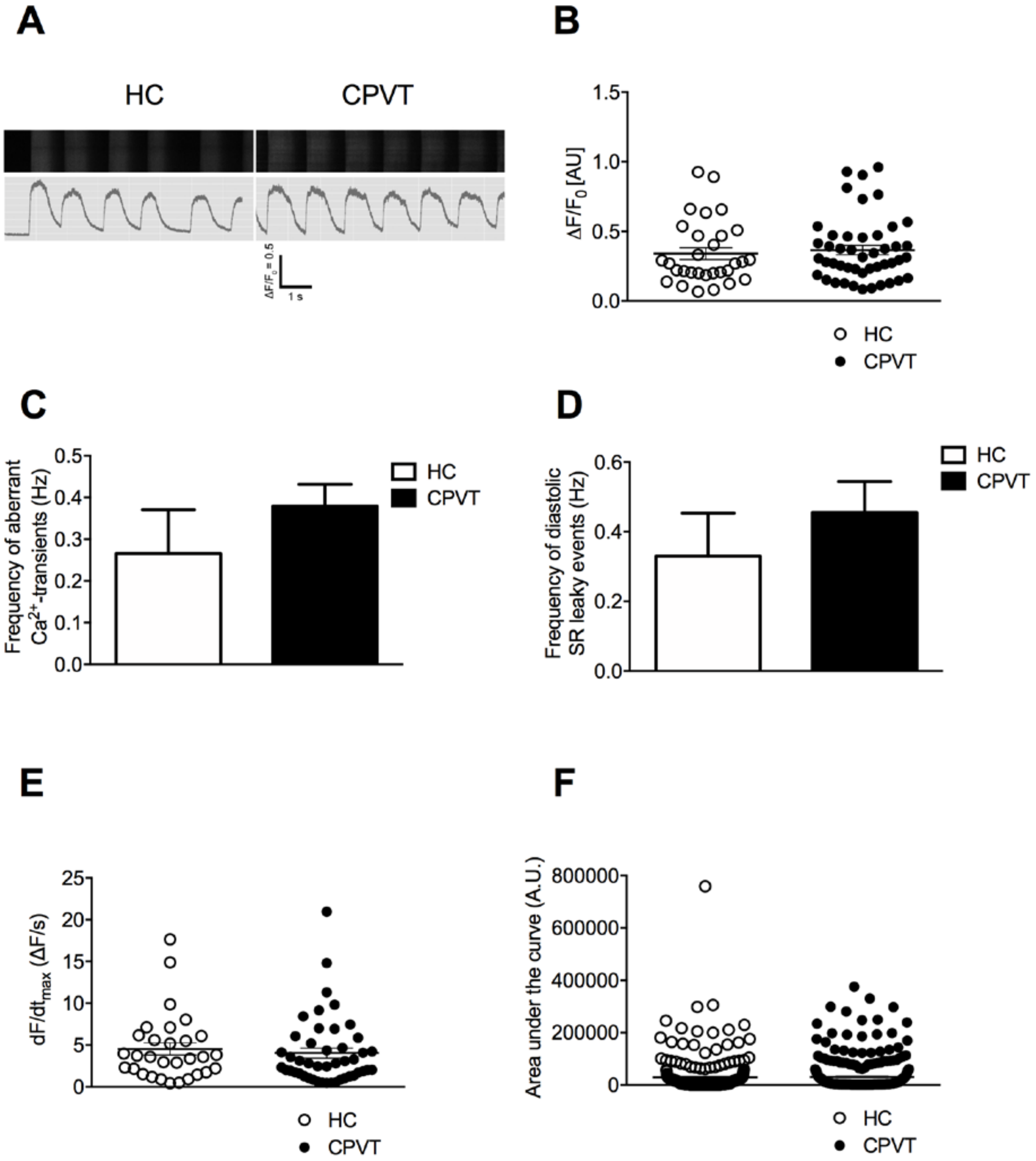
A
HC
CPVT
ΔF/F0 = 0.5
1 s
B
ΔF/F0 [AU]
1.5
1.0
0.5
0.0
HC
CPVT
C
Frequency of aberrant Ca2+-transients (Hz)
0.5
0.4
0.3
0.2
0.1
0.0
HC
CPVT
D
Frequency of diastolic SR leaky events (Hz)
0.6
0.4
0.2
0.0
HC
CPVT
E
dF/dtmax (ΔF/s)
25
20
15
10
5
0
HC
CPVT
F
Area under the curve (A.U.)
800000
600000
400000
200000
0
HC
CPVT

Figure S7

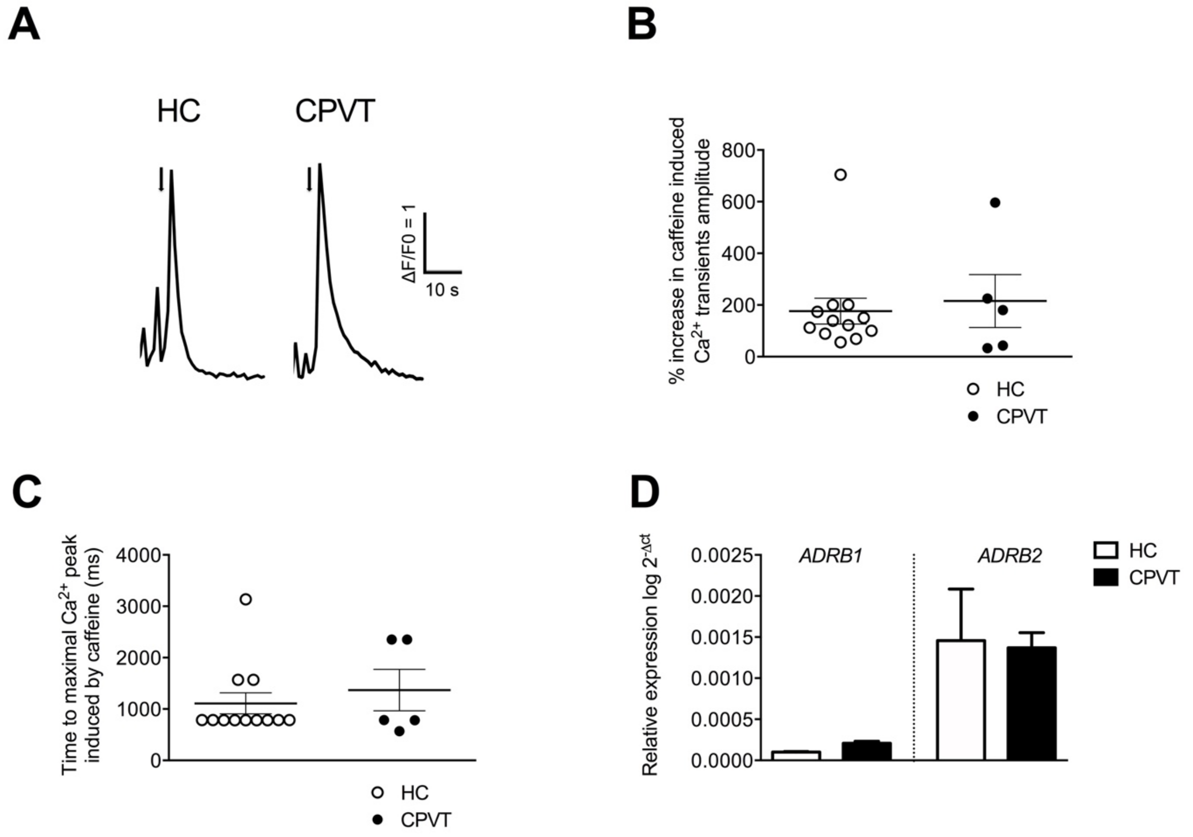
A
HC
CPVT
ΔF/F0 = 1
10 s
B
% increase in caffeine induced Ca2+ transients amplitude
800
600
400
200
0
HC
CPVT
C
Time to maximal Ca2+ peak induced by caffeine (ms)
4000
3000
2000
1000
0
HC
CPVT
D
Relative expression log 2-Δct
0.0025
0.0020
0.0015
0.0010
0.0005
0.0000
ADRB1
ADRB2
HC
CPVT

Figure S8

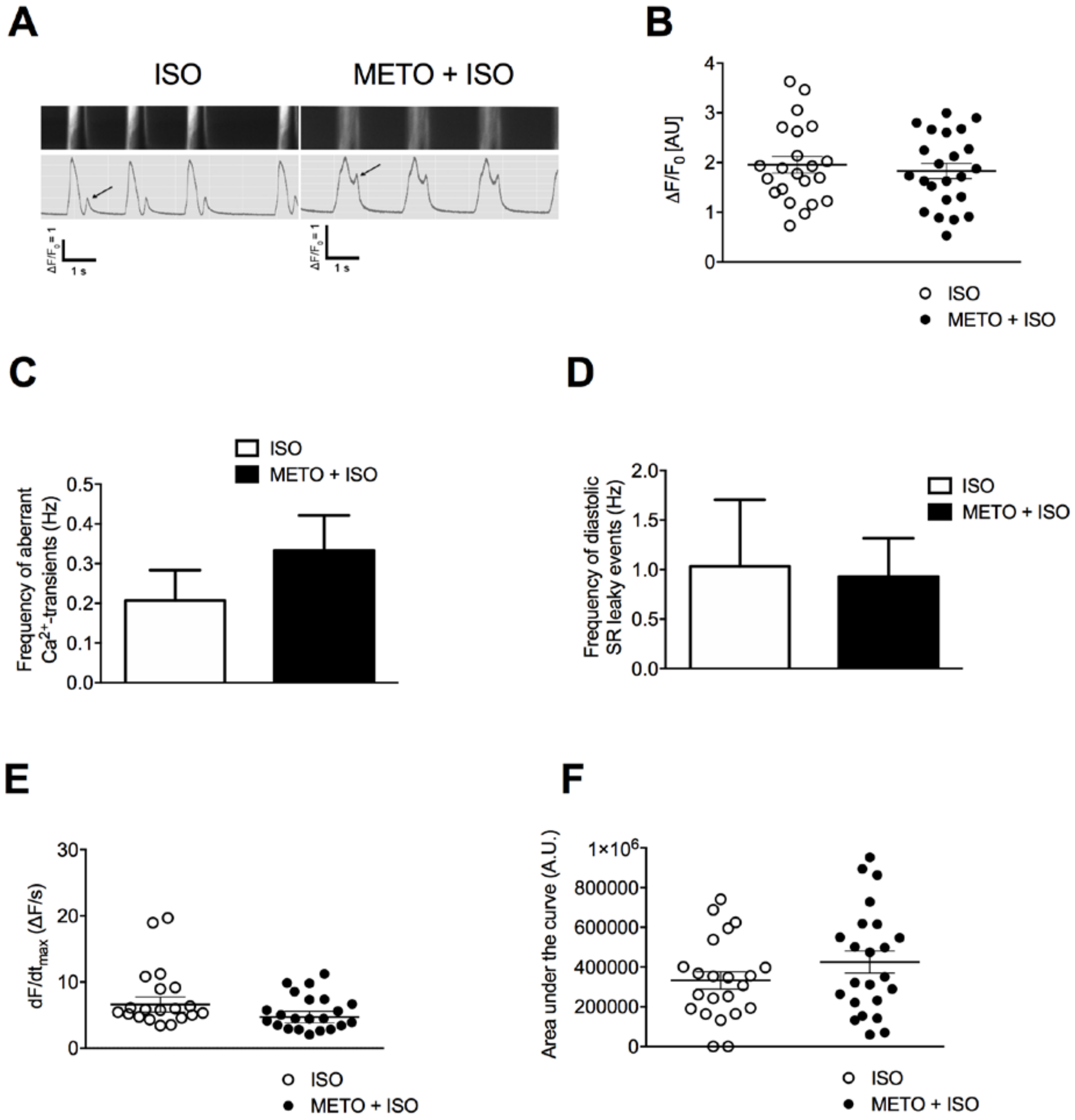
A
ISO
METO + ISO
ΔF/F0 = 1
1 s
B
ΔF/F0 [AU]
ISO
METO + ISO
C
Frequency of aberrant Ca2+-transients (Hz)
ISO
METO + ISO
D
Frequency of diastolic SR leaky events (Hz)
ISO
METO + ISO
E
dF/dtmax (ΔF/s)
ISO
METO + ISO
F
Area under the curve (A.U.)
ISO
METO + ISO

Figure S9

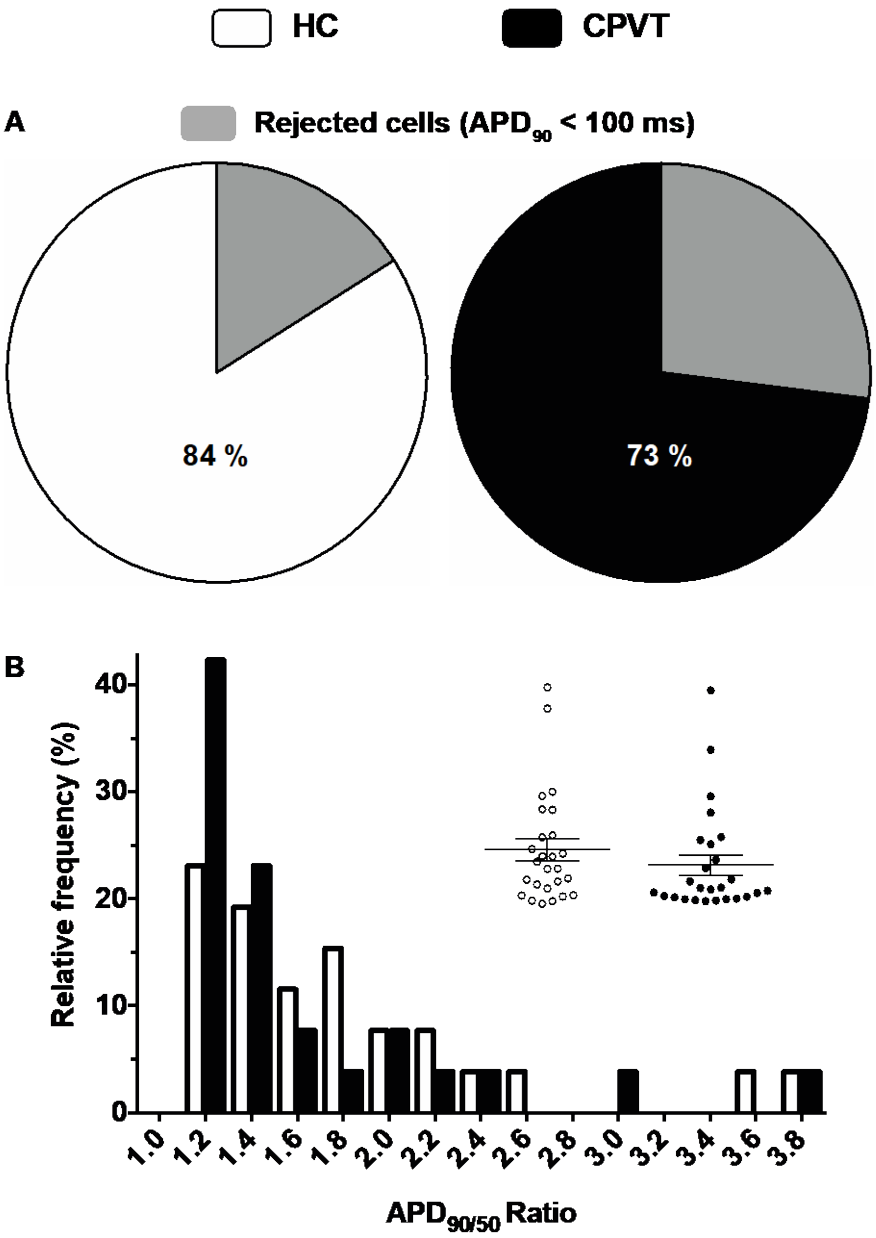
HC
CPVT
A
Rejected cells (APD90 < 100 ms)
84 %
73 %
B
Relative frequency (%)
40
30
20
10
0
1.0
1.2
1.4
1.6
1.8
2.0
2.2
2.4
2.6
2.8
3.0
3.2
3.4
3.6
3.8
APD90/50 Ratio

Figure S10

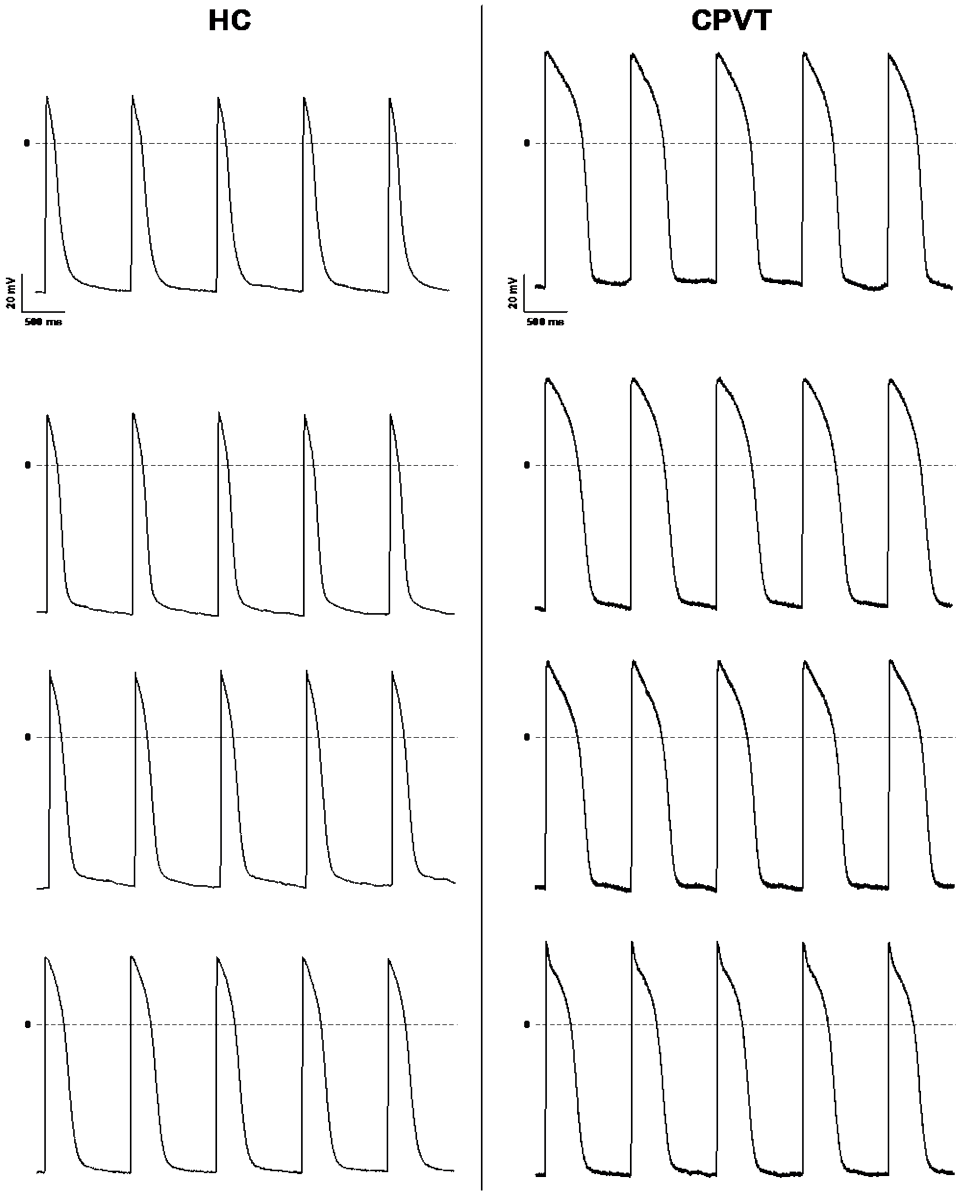

Figure S11

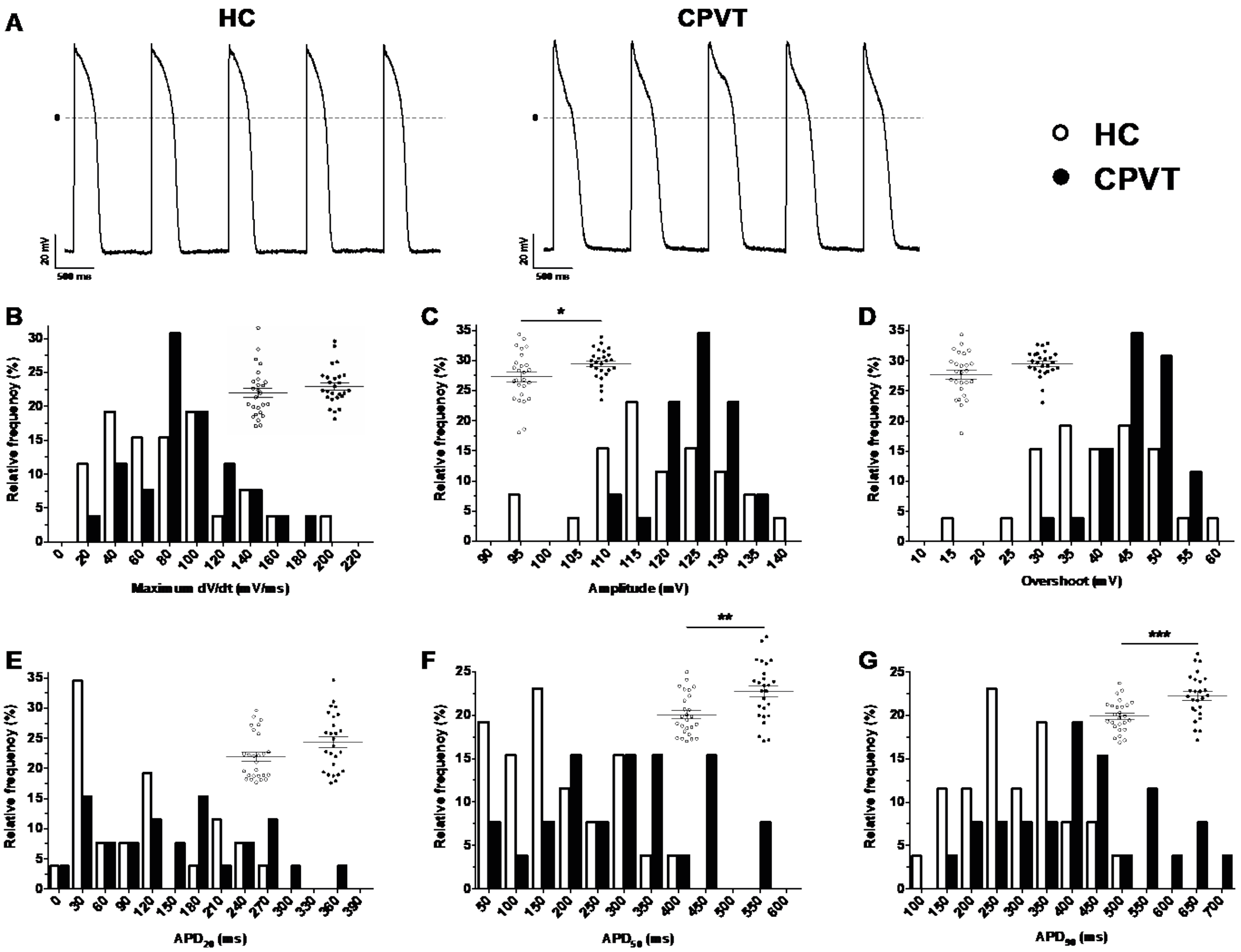
A
HC
CPVT
20 mV
500 ms
HC
CPVT
B
Relative frequency (%)
Maximum dV/dt (mV/ms)
C
Amplitude (mV)
D
Overshoot (mV)
E
APD20 (ms)
F
APD50 (ms)
G
APD90 (ms)

Figure S12

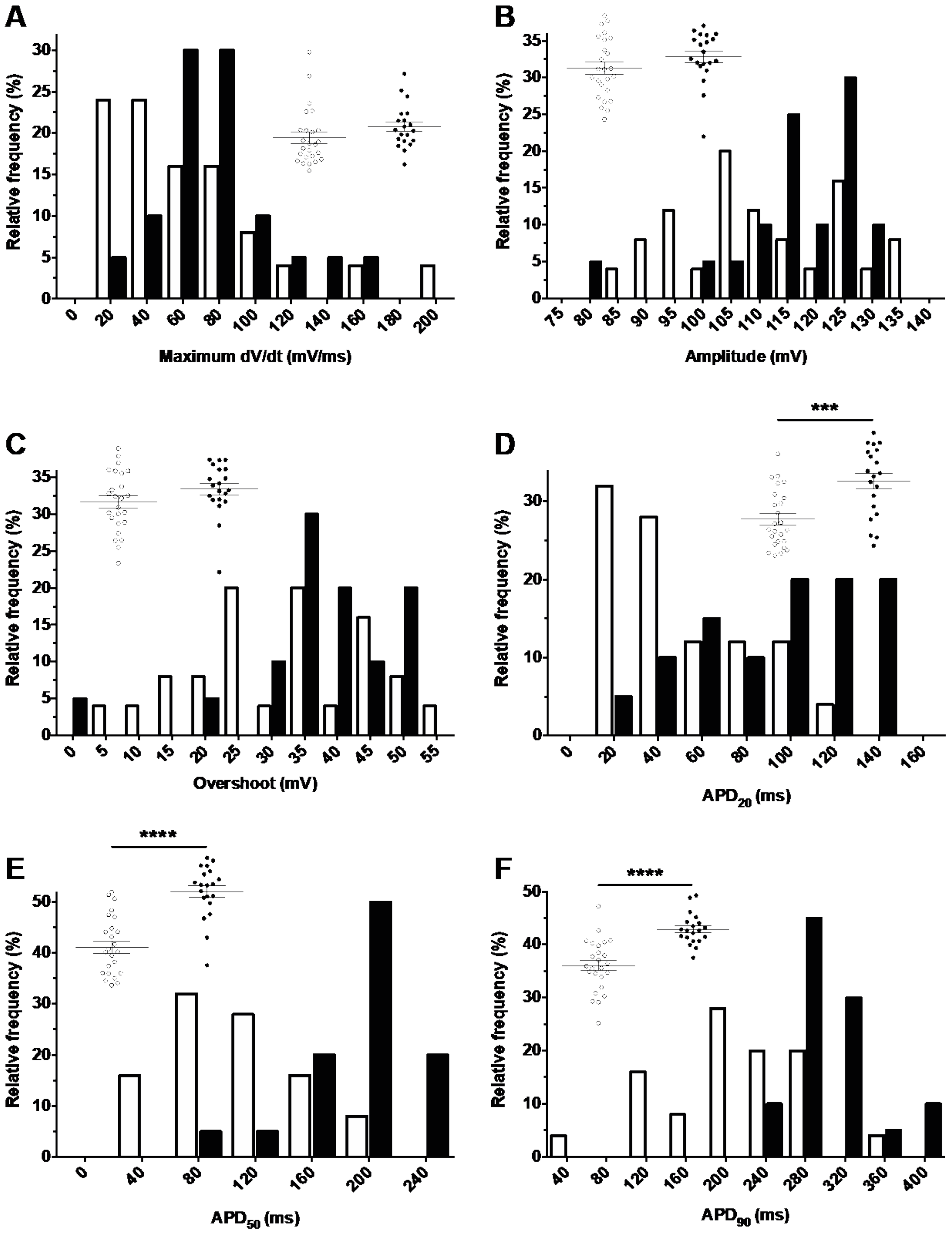
A
Relative frequency (%)
0 20 40 60 80 100 120 140 160 180 200
Maximum dV/dt (mV/ms)
B
Relative frequency (%)
75 80 85 90 95 100 105 110 115 120 125 130 135 140
Amplitude (mV)
C
Relative frequency (%)
0 5 10 15 20 25 30 35 40 45 50 55
Overshoot (mV)
D
***
Relative frequency (%)
0 20 40 60 80 100 120 140 160
APD$_{20}$ (ms)
E
****
Relative frequency (%)
0 40 80 120 160 200 240
APD$_{50}$ (ms)
F
****
Relative frequency (%)
40 80 120 160 200 240 280 320 360 400
APD$_{90}$ (ms)

Figure S13

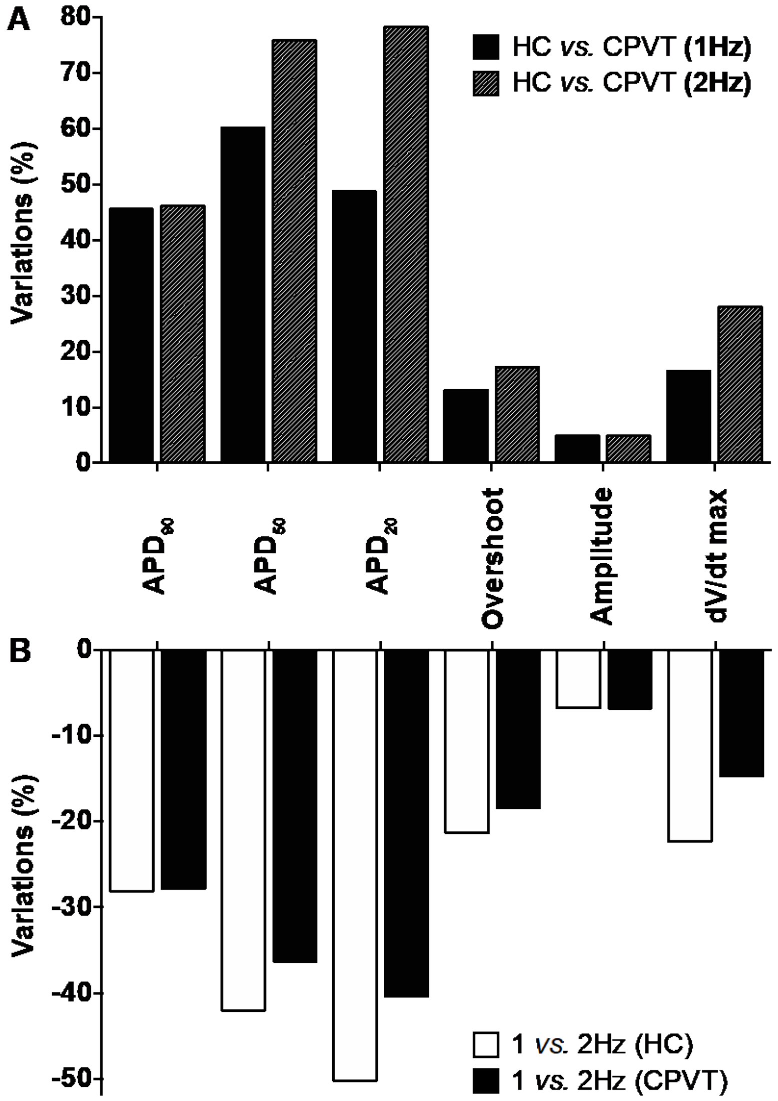
A
80
70
60
50
40
30
20
10
0
Variations (%)
HC vs. CPVT (1Hz)
HC vs. CPVT (2Hz)
APD90
APD50
APD20
Overshoot
Amplitude
dV/dt max
B
0
-10
-20
-30
-40
-50
Variations (%)
1 vs. 2Hz (HC)
1 vs. 2Hz (CPVT)

References

1. Peng, W.; Shen, H.; Wu, J.; Guo, W.; Pan, X.; Wang, R.; Chen, S.R.; Yan, N. Structural basis for the gating mechanism of the type 2 ryanodine receptor RyR2. LID - aah5324 [pii]. *Science* **2016**, *354*.
2. E Dayringer, H.; Tramontano, A.; R Sprang, S.; J Fletterick, R. Interactive program for visualization and modelling of proteins, nucleic acids and small molecules. *Journal of Molecular Graphics* **1986**, *4*, 82-87, doi:https://doi.org/10.1016/0263-7855(86)80002-9.
3. Qu, C.; Puttonen, K.A.; Lindeberg, H.; Ruponen, M.; Hovatta, O.; Koistinaho, J.; Lammi, M.J. Chondrogenic differentiation of human pluripotent stem cells in chondrocyte co-culture. *Int J Biochem Cell Biol* **2013**, *45*, 1802-1812, doi:10.1016/j.biocel.2013.05.029.
4. Pesl, M.; Acimovic, I.; Pribyl, J.; Hezova, R.; Vilotic, A.; Fauconnier, J.; Vrbsky, J.; Kruzliak, P.; Skladal, P.; Kara, T., et al. Forced aggregation and defined factors allow highly uniform-sized embryoid bodies and functional cardiomyocytes from human embryonic and induced pluripotent stem cells. *Heart and vessels* **2014**, *29*, 834-846, doi:10.1007/s00380-013-0436-9.
5. Pesl, M.; Pribyl, J.; Acimovic, I.; Vilotic, A.; Jelinkova, S.; Salykin, A.; Lacampagne, A.; Dvorak, P.; Meli, A.C.; Skladal, P., et al. Atomic force microscopy combined with human pluripotent stem cell derived cardiomyocytes for biomechanical sensing. *Biosens Bioelectron* **2016**, *85*, 751-757, doi:10.1016/j.bios.2016.05.073.
6. Kunova, M.; Matulka, K.; Eiselleova, L.; Trckova, P.; Hampl, A.; Dvorak, P. Development of humanized culture medium with plant-derived serum replacement for human pluripotent stem cells. *Reproductive biomedicine online* **2010**, *21*, 676-686, doi:10.1016/j.rbmo.2010.06.027.
7. Moreau, A.; Mercier, A.; Theriault, O.; Boutjdir, M.; Burger, B.; Keller, D.I.; Chahine, M. Biophysical, Molecular, and Pharmacological Characterization of Voltage-Dependent Sodium Channels From Induced Pluripotent Stem Cell-Derived Cardiomyocytes.
